# Splicing is an alternate oncogenic pathway activation mechanism in glioma

**DOI:** 10.1038/s41467-022-28253-4

**Published:** 2022-01-31

**Authors:** Robert Siddaway, Scott Milos, Arun Kumaran Anguraj Vadivel, Tara H. W. Dobson, Jyothishmathi Swaminathan, Scott Ryall, Sanja Pajovic, Palak G. Patel, Javad Nazarian, Oren Becher, Michael Brudno, Arun Ramani, Vidya Gopalakrishnan, Cynthia Hawkins

**Affiliations:** 1 Arthur and Sonia Labatt Brain Tumour Research Centre, The Hospital for Sick Children, Toronto, Canada; 2 Department of Pediatrics - Research, The University of Texas MD Anderson Cancer Center, Houston, TX 77030 USA; 3 Department of Integrative Systems Biology, Children’s National Medical Center, George Washington University, Washington, DC 20010 USA; 4 Department of Oncology, University Children’s Hospital, Zurich, Switzerland; 5 Ann & Robert H. Lurie Children’s Hospital of Chicago, Chicago, IL 60611 USA; 6 Department of Computer Science, University of Toronto, Toronto, Canada; 7 Centre for Computational Medicine, The Hospital for Sick Children, Toronto, Canada; 8 University Health Network, Toronto, ON Canada; 9 Department of Laboratory Medicine and Pathobiology, University of Toronto, Toronto, Canada; 10 Division of Pathology, The Hospital for Sick Children, Toronto, Canada

**Keywords:** Paediatric cancer, RNA splicing, Cancer genomics, CNS cancer

## Abstract

High-grade diffuse glioma (HGG) is the leading cause of brain tumour death. While the genetic drivers of HGG have been well described, targeting these has thus far had little impact on survival suggesting other mechanisms are at play. Here we interrogate the alternative splicing landscape of pediatric and adult HGG through multi-omic analyses, uncovering an increased splicing burden compared with normal brain. The rate of recurrent alternative splicing in cancer drivers exceeds their mutation rate, a pattern that is recapitulated in pan-cancer analyses, and is associated with worse prognosis in HGG. We investigate potential oncogenicity by interrogating cancer pathways affected by alternative splicing in HGG; spliced cancer drivers include members of the RAS/MAPK pathway. RAS suppressor *neurofibromin 1* is differentially spliced to a less active isoform in >80% of HGG downstream from REST upregulation, activating the RAS/MAPK pathway and reducing glioblastoma patient survival. Overall, our results identify non-mutagenic mechanisms by which cancers activate oncogenic pathways which need to accounted for in personalized medicine approaches.

## Abstract (web summary)

Targeting genetic drivers of high grade diffuse glioma (HGG) has not improved patient survival, suggesting the involvement of other mechanisms. Here, across cancer types, the authors identify increased alternative splicing burden in cancer drivers compared to mutation rate as an alternative mechanism for activation of oncogenic pathways such as RAS/MAPK.

## Introduction

The genetic drivers of high-grade glioma (HGG) have been well-described, with isocitrate dehydrogenase (*IDH*) mutations and epidermal growth-factor receptor (*EGFR*) amplifications common in adult diffuse glioma, while pediatric HGG (pHGG) harbor high-frequency mutations in histones H3.1 and H3.3 as well as platelet-derived growth-factor receptor alpha (*PDGFRA*) alterations^1–8^. Some events are shared between pediatric and adult HGG; for example, *neurofibromin 1* (*NF1*) is mutated in 5% of all cancers and 10% of HGG^3,4,9^ However, the prognosis for both children and adults diagnosed with this devastating disease remains dismal. The oncogenicity of DNA mutations has been well studied but is imperfect in its ability to predict tumor behavior and therapeutic response, suggesting other mechanisms, including epigenetic, transcriptomic, and proteomic, are at play. Alternative splicing (AS), which increases transcript diversity and has been shown to be altered in adult cancer^10^, has the potential to affect cancer genes through removal or alteration of protein domains and post-translational modifications (PTM), nonsense-mediated decay, or protein truncation.

Here, we find that AS more frequently targets cancer genes than do point mutations across a broad spectrum of cancers, a phenomenon that is particularly striking in HGG where cancer-driver splicing, but not mutation, burden is significantly associated with survival. Characterization of the HGG AS landscape demonstrates a convergence on cancer-driver genes. To test the potential for a functional role of these AS events, we investigate the role of alternative splicing of *NF1*. We found preferential inclusion of *NF1* exon23a in HGG increased RAS/MAPK activity independent of other RAS/MAPK alterations and was associated with worse patient survival. *NF1*^3,4,9^, is differentially spliced in over 80% of HGG, indicating a much broader, NF1-mediated, RAS-activating event than by mutation alone. Overall, our data demonstrate an alternative, clinically relevant, mechanism of oncogenic pathway activation in cancer that will be important to integrate into future therapeutic workflows for optimal precision medicine.

## Results

### Spliceosome mutations are associated with increased alternative splicing in pHGG

**Fig. 1:**
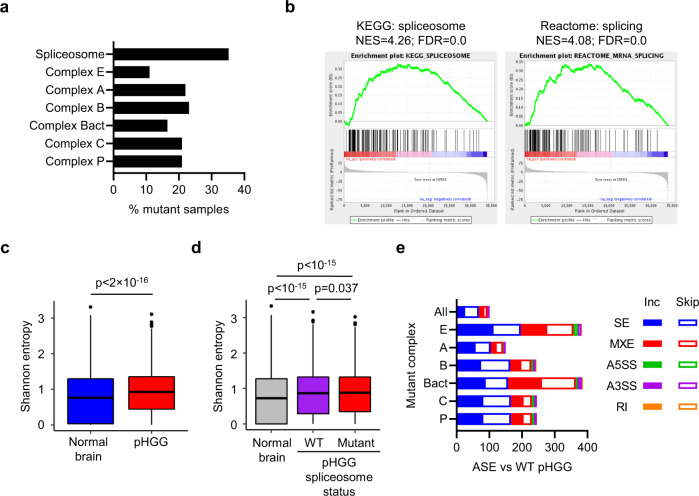
The landscape of alternative splicing in pHGG. **a** Frequency of spliceosome mutations in pHGG in total (Spliceosome) and by subcomplex. **b** Gene set enrichment analysis (GSEA) comparing pHGG (*n* = 64) and normal brain (*n* = 20) for spliceosome components and mRNA splicing pathway. NES normalized enrichment score, FDR false discovery rate. **c** Splicing burden, as measured by the median Shannon entropy of multi-isoform genes, was plotted for normal brain (*n* = 20) and pHGG (*n* = 64). The box shows the interquartile range (IQR), the line shows the median, and the whiskers extend to 1.5×IQR. **d** Splicing burden was plotted for normal brain (*n* = 20) plus pHGG with wild-type (WT, *n* = 24) or mutant (*n* = 28) spliceosomes. The box shows the interquartile range (IQR), the line shows the median, and the whiskers extend to 1.5×IQR. **e** Differential splicing events (ASE; splice change|Δψ|>0.15, FDR < 0.05) were identified in pHGG with mutations in each spliceosome complex relative to pHGG with WT spliceosomes. Each bar shows the number of each type of event that was preferentially included (Inc) or skipped in mutant pHGG. SE skipped exon, MXE mutually exclusive exon, A5SS alternative 5’ splice site, A3SS alternative 3’ splice site, RI retained intron (see Supplementary Fig. S2a for schematic). Statistical tests: Wilcoxon rank-sum test (**c**), Pairwise Wilcoxon rank-sum tests with Benjamini–Hochberg correction (**d**). Source data are provided as a Source Data file.

Investigating the mutational landscape of our pHGG cohort, we found that 34% of patients (*n* = 31/91) had at least one spliceosome mutation including single nucleotide variants or copy number alterations (Fig. 1a, Supplementary Fig. S1a, and Supplementary Data 1 and 2). In an independent pHGG cohort^11,12^, 28% (40/141) of patients had spliceosome mutations (Supplementary Fig. S1b). The spliceosome is composed of multiple subcomplexes, with many proteins involved in one or more subcomplexes (Supplementary Data 2). Core components that are used throughout the spliceosome lifecycle were the most frequently mutated, with 11–23% of patients (*n* = 10–21) having a mutation in any complex (Fig. 1a and Supplementary Fig. S1c). Spliceosome mutations in genes including *SF3B1*, *U2AF*, and *SRSF2* frequently occur in other tumors such as leukemia and myelodysplastic syndrome^13^, while highly recurrent alterations in the U1 snRNA were recently identified in multiple cancers, including medulloblastoma^14,15^. Hotspot mutations in these genes and the U1 snRNA alterations were not identified in our cohort (Supplementary Data 2).

Given the frequent spliceosome mutations, we hypothesized that pHGG may be prone to disrupted AS. In RNA-Seq analysis comparing 64 pHGG (*n* = 64) with the normal brain (*n* = 20), splicing pathways were upregulated (Fig. 1b and Supplementary Data 1), irrespective of direct mutations in the spliceosome and not related to differential DNA methylation (Supplementary Fig. S1f). An upregulation of splicing pathways was also seen in adult diffuse glioma using RNA-Seq from The Cancer Genome Atlas (TCGA; IDH-wt (*n* = 243), IDH-mut/non-codel (*n* = 270), IDH-mut/codel (*n* = 173), normal brain (*n* = 5)) and (*P* ≤ 0.005; Supplementary Fig. S1d, e) suggesting this may be a general glioma phenomenon.

In keeping with the upregulation of splicing pathways, analysis of alternative splicing events (ASE) (rMATS^16^), found 247,507 ASE across the dataset (Supplementary Fig. S2a, b) with 1827 differential ASE from 904 genes identified in pHGG compared with a normal brain. 844 differential ASE (absolute Δψ > 0.15, FDR < 0.05) associated with mutant spliceosomes, which were mostly skipped exons or mutually exclusive exons and unique to tumors mutated in a given complex (Fig. 1e and Supplementary Fig. S1j, k). Subcomplexes E (the earliest assembly stage) and Bact (the subcomplex where the catalytic core is fully assembled) had the biggest effect on AS compared with spliceosome-WT tumors (Fig. 1a, e and Supplementary Fig. S1k).

Globally, pHGG and normal brain could be efficiently separated in unsupervised hierarchical clustering and t-SNE analyses based on ASEs (Supplementary Fig. S1g, h). We then quantified alternative splicing in pHGG compared with the normal brain. First, we found that splice junction usage was more variable (*P* < 2 × 10^−16^; Supplementary Fig. S1i). Next, we calculated the Shannon entropy of each multi-isoform gene^17^, finding a significant increase in pHGG indicating that more isoforms are expressed than in the normal brain, irrespective of spliceosome-mutant status (pHGG as a whole vs normal brain *P* < 2 × 10^−16^; spliceosome-WT pHGG vs. normal brain *P* = 6 × 10^−11^; spliceosome-mutant pHGG vs. normal brain *P* < 10^−15^; spliceosome-WT vs. spliceosome-mutant pHGG *P* = 0.037, Fig. 1c, d).

### Cancer-driver genes are enriched among differential splicing events in pHGG

**Fig. 2:**
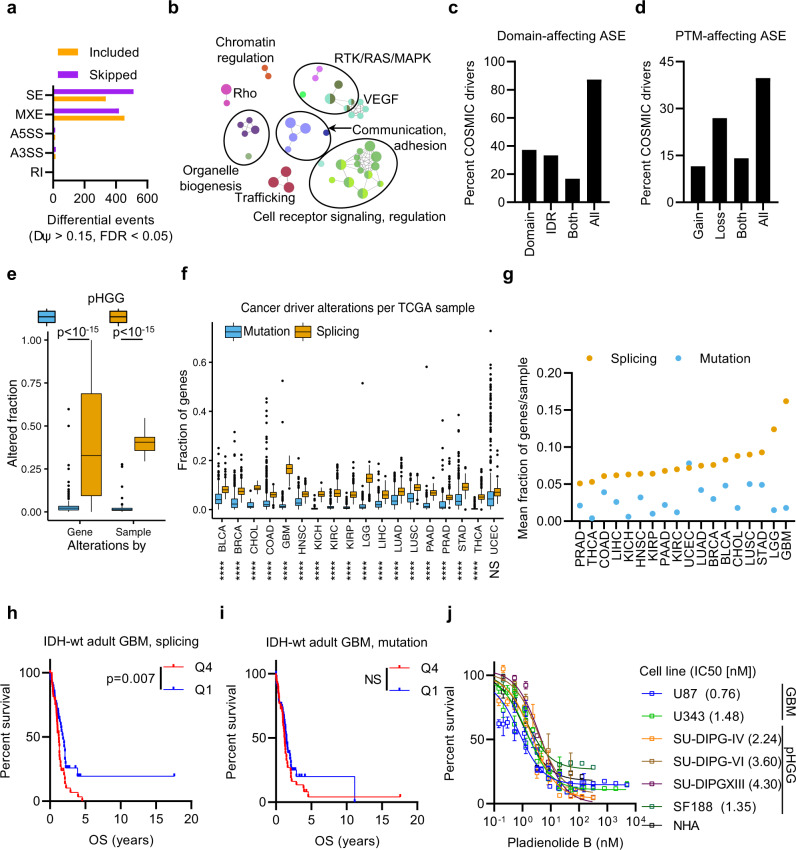
Pan-cancer alternative splicing converges on cancer-driver genes. **a** The number of differential ASE that are preferentially included or skipped in pHGG from each ASE category. SE skipped exon, MXE mutually exclusive exon, A5SS alternative 5’ splice site, A3SS alternative 3’ splice site, RI retained intron (see Supplementary Fig. S2a for schematic). **b** Reactome pathways enriched among genes differentially spliced in pHGG at false discovery rate (FDR) < 0.05. **c** Percentage of COSMIC cancer-driver genes with differential ASE predicted to affect structural domains or intrinsically disordered domains (IDR) mapped in UniProt. **d** Percentage of COSMIC cancer-driver genes with differential ASE predicted to affect post-translational modification (PTM) sites mapped in UniProt. **e** Burden of splicing and mutations among COSMIC cancer-driver genes in pHGG (*n* = 51). The left group (Gene) shows the fraction of samples with an alteration per driver gene. The right group (Sample) shows the fraction of driver genes that are altered per sample. The box marks the interquartile range (IQR) and shows the median value. The whiskers extend to 1.5× IQR and outliers outside this are plotted separately. **f** Pan-cancer burden of splicing and mutations in COSMIC cancer-driver genes, showing the fraction of driver genes that are altered per sample. The box marks the interquartile range (IQR) and shows the median value. The whiskers extend to 1.5× IQR and outliers outside this are plotted separately. See Supplementary Data 4 for *n* and *P* values. **g** Data in (**f**) are summarized to show the mean fraction of altered genes per sample across each tumor type. **h** Kaplan–Meier survival plot of IDH-wt GBM patients that are in the top (Q4, *n* = 55) or bottom (Q1, *n* = 57) quartile of splicing burden of cancer-driver genes. **i** Kaplan–Meier survival plot of IDH-wt GBM patients that are in the top (Q4, *n* = 55) or bottom (Q1, *n* = 56) quartile of mutation burden of cancer-driver genes. **j** Cell lines were treated with increasing concentration of pladienolide B for 5 days and viability was measured with alamarBlue (*n* = 6). Bars show mean ± standard error. Statistical tests: *t* (**e**) with Benjamini–Hochberg multiple hypothesis testing correction (**f**), log-rank (**h**, **i**). ****P < 0.0001. NS not significant. Source data are provided as a Source Data file.

In total, 1827 differential ASE from 904 genes were identified in pHGG, predominantly skipped exon and mutually exclusive exon events, (Fig. 2a and Supplementary Fig. S2a–c). We validated select targets by RT-PCR in pHGG/normal brain pairs (Supplementary Fig. S2d). In all, 79% of spliced genes were not also differentially expressed, indicating the differential ASE we identified are not expression-level artifacts (Supplementary Fig. S2e). To confirm our findings, we compared the targets identified by rMATS^16^ with a second algorithm, SUPPA2^18^, which estimates splicing changes from transcript abundances rather than junction counts and thus provides a very different method of analyzing the splicing landscape of pHGG. SUPPA2 identified 2576 events from 1482 genes, with a highly significant overlap in differentially spliced genes between the two approaches (*P* = 10^−120^; Supplementary Fig. S2f). Interestingly, although large numbers of intron retentions have recently been described in adult GBM^10,19^, neither rMATS (6) or SUPPA2 (30) identified large numbers of significantly differential intron retentions when comparing pHGG with normal brain despite identifying over 4000 intron retention events across the dataset. This could be algorithm-related, however, as using SplAdder, the algorithm used in the adult GBM publication^10^, identified >3× more (13,866) RI events, and determined 565 to be significant (94 and 20× more than rMATS/SUPPA2, respectively). Importantly, three genes in which we confirmed differential splicing in pHGG using RT-PCR (*FGFR1*, *MBD1*, *SMARCC2*) were identified only with rMATS. Given that rMATS could identify additional true splicing events beyond SUPPA2, while appearing more conservative overall in terms of events identified, we proceeded with the 1827 differential ASE we identified in pHGG using rMATS for subsequent analyses.

We found that the 1827 ASE in pHGG were differentially spliced regardless of the spliceosome mutation status. In addition, spliceosome-mutant pHGG have an increased burden of ASEs, which varied with the component of the spliceosome complex that was mutant (Fig. 1e and Supplementary Fig. S1k).

Differentially spliced genes were enriched in the RAS/MAPK (*P* < 2 × 10^−4^), chromatin (*P* = 1.3 × 10^−4^), cellular communication (*P* < 3 × 10^−4^), and trafficking (*P* = 3 × 10^−9^) pathways (Fig. 2b), with very similar findings among the genes identified by SUPPA2 (Supplementary Fig. S2g). Remarkably, genes in the Catalogue of Somatic Mutations in Cancer (COSMIC) cancer-driver census^20^ were enriched significantly more than expected (*P* = 3 × 10^−26^), with 78 genes (8%; 185 ASE) differentially spliced in pHGG, in particular from the RAS/MAPK and chromatin pathways (Supplementary Fig. S3a, b). Furthermore, among oncogenic pathways curated by TCGA^9^, the RTK/RAS/MAPK and PI3K pathways were significantly affected by splicing but not by expression (Supplementary Fig. S3c). The majority of differential ASE in cancer-driver genes were predicted to affect protein domains (53%), intrinsically disordered regions (50%) or PTMs (45%; Fig. 2c, d).

Collectively, these data imply that pHGG frequently activates oncogenic processes non-mutationally. Accordingly, the rate of recurrent cancer-driver splicing was significantly higher than mutation in pHGG within both individual samples and genes (both *P* < 10^−15^; Fig. 2e). To test if the frequency of AS, and the convergence on cancer drivers, was a more general phenomenon, we used TCGA data for 18 tumor types with available normal tissue RNA-Seq. Remarkably, 17 had significantly higher cancer-driver splicing burden per sample than mutations (*P* ≤ 10^−17^), with most also having more recurrent AS alterations than mutations (Fig. 2f and Supplementary Fig. S3d, e).

Adult diffuse gliomas (glioblastoma, GBM; lower-grade glioma; LGG) had the highest level of splice-driven alterations (Fig. 2f, g). Those with the highest differential cancer-driver splicing had significantly worse overall survival (OS) (*P* < 10^−15^) while those with higher cancer-driver mutation burdens did not (Supplementary Fig. S4a, b). Adult diffuse glioma occurs in three main subgroups defined by isocitrate dehydrogenase (IDH) mutations and co-deletion of chromosomes 1p and 19q^4^. IDH wild-type (IDH-wt) GBM have poor outcome compared with IDH-mutant diffuse glioma (with (IDH-mut/codel) or without (IDH-mut/non-codel) 1p/19q co-deletion)^4^, and GBM had increased cancer-driver AS (Supplementary Fig. S4c). However, on multivariate survival analysis the effect of cancer-driver AS on OS was subgroup-independent (*P* = 0.01; Supplementary Fig. S4d). Splicing, but not mutation burden, was significantly associated with worse OS for IDH-wt GBM (*P* = 0.007) and IDH-mut/non-codel astrocytoma (*P* = 0.015), with a trend (*P* = 0.18) for IDH-mut/codel oligodendroglioma (Fig. 2h, i and Supplementary Fig. S4e–i).

To test the dependence of HGG on the spliceosome, we treated both pHGG, GBM, and normal human astrocyte cell lines with the spliceosome inhibitor pladienolide B. All lines including normal human astrocyte controls were exquisitely sensitive to this drug (IC50 0.76–4.30 nM; Fig. 2j). Similar results were obtained with a second inhibitor, madrasin (Supplementary Fig. S4j), demonstrating that, although unlikely to be directly targetable, HGG cells have a fundamental requirement for spliceosome activity to remain viable.

### Functional consequences of chromatin regulator disruption by alternative splicing

While the frequency of AS in cancer genes and its association with more aggressive tumor behavior are intriguing, an important question is the potential for functional effects of these splicing events. We therefore looked more closely at the functional implications of the chromatin modifier and RAS/MAPK pathway ASEs we found in HGG.

**Fig. 3:**
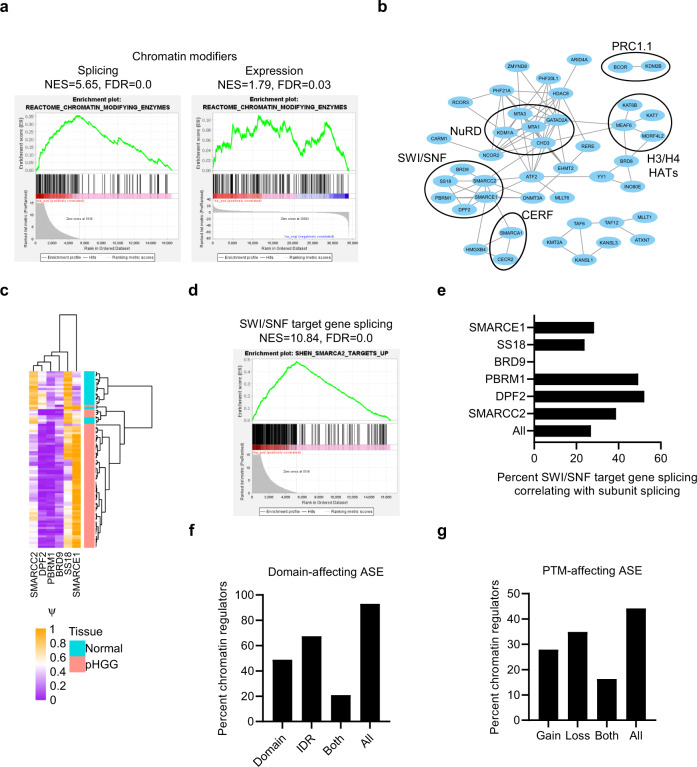
Chromatin regulators are disrupted by alternative splicing in pHGG. **a** Gene set enrichment analysis of chromatin-modifying enzymes among genes differentially spliced (left) or differentially expressed (right) in pHGG. NES normalized enrichment score, FDR false discovery rate. **b** Protein–protein interactions between chromatin-related proteins that are differentially spliced in pHGG were retrieved from STRING. Edge thickness represents interaction confidence. SWI/SNF mammalian switch/sucrose-nonfermentable, NuRD nucleosome remodeling and deacetylase, PRC1.1 polycomb-repressive complex 1.1, CERF CECR2-containing remodeling factor complex. **c** Heatmap of percent inclusion (ψ) of differential splicing events (|Δψ| >0.15, FDR < 0.05) identified between pHGG (*n* = 64) and normal brain (*n* = 20) in SWI/SNF complex members. **d** Gene set enrichment analysis (GSEA) of SWI/SNF target gene enrichment among genes differentially spliced in pHGG. NES normalized enrichment score, FDR false discovery rate. **e** Proportion of SWI/SNF target genes whose ψ correlates significantly (Benjamini–Hochberg corrected *P* < 0.05) with ψ of each SWI/SNF complex member across pHGG samples (*n* = 64), or with the maximally variant complex member (“All”) in each sample. **f** Percentage of chromatin regulators with differential ASE predicted to affect structural domains and intrinsically disordered domains (IDR) mapped in UniProt. **g** Percentage of chromatin regulators with differential ASE predicted to affect post-translational modification (PTM) sites mapped in UniProt. Statistical tests: *t* test with Benjamini–Hochberg multiple hypothesis testing correction (**e**). Source data are provided as a Source Data file.

Chromatin disruption through recurrent H3K27M mutations and DNA hypomethylation have been characterized in pHGG^1,2,21^. Our data suggest AS may also affect epigenetic regulation of pHGG, with chromatin modifiers more affected by splicing than expression changes (Figs. 2b and 3a). Strikingly, among the 48 differentially spliced chromatin regulators, several protein complexes had multiple affected members, in particular mammalian switch/sucrose-nonfermentable (SWI/SNF), nucleosome remodeling and deacetylase (NuRD), and polycomb-repressive complex 1.1 (PRC1.1; Fig. 3b, c). We validated two, MBD1 and SMARCC1, by RT-PCR (Supplementary Fig. S2d). Interestingly, the SWI/SNF complex has previously been implicated in alternative splicing of its target genes^22^, which were also enriched in pHGG differential ASE, with high proportions correlating significantly with the splicing of individual complex members (Fig. 3d, e). These data implicate AS in epigenetic regulation at the level of chromatin regulators and their downstream target genes.

To further investigate this, we analyzed the predicted effects of differential ASE in chromatin regulators. Most (95%) of the AS involved protein domains (49%), intrinsically disordered regions (67%) and/or PTMs (44%), suggesting that AS in chromatin regulators will affect their function (Fig. 3f, g).

### Activation of RAS/MAPK signaling by an *NF1* isoform switch in HGG

**Fig. 4:**
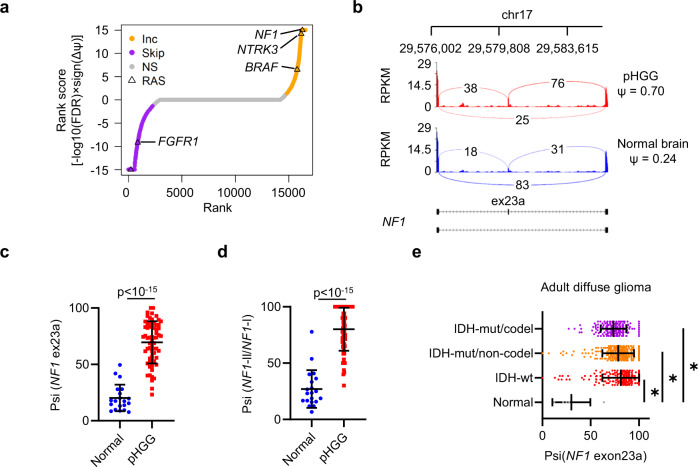
Alternative splicing of *NF1* exon23a in HGG. **a** Genes were ranked according to their most significant splicing score. Significance is shown by point color (orange, inclusion [Inc]; purple, skipping; NS not significant). RAS/MAPK pathway genes with significant differential ASE (ASE; | Δψ | >0.15, FDR < 0.05) are marked. **b** Sashimi plot showing average reads per kilobase per million (RPKM) in pHGG and normal brain for coverage of the *NF1* exon23a skipped exon and immediate up- and downstream exons. **c** *NF1* exon23a percent inclusion (psi) in pHGG (*n* = 64) and normal brain (*n* = 20) as determined by junction coverage. Bars show mean ± standard deviation. **d** *NF1* exon23a inclusion in pHGG and normal brain was determined from the ratio of isoform expression of *NF1*-I and *NF1*-II transcripts in pHGG (*n* = 64) and normal brain (*n* = 20) as determined by junction coverage. Bars show mean ± standard deviation. **e** *NF1* exon23a percent inclusion (psi) based on NF1 transcript ratios in TCGA diffuse glioma dataset: adult normal brain (*n* = 5) and IDH-wt (*n* = 231; *P* = 0.01), IDH-mut/non-codel (*n* = 256; *P* = 0.01) and IDH-mut/codel (*n* = 170; *P* = 0.02) HGG. Bars show mean ± standard deviation. Statistical tests: *t* (**c**, **d**), Brown–Forsythe ANOVA with Dunnett’s T3 multiple comparisons test (**e**). *P < 0.05. Source data are provided as a Source Data file.

RTK/RAS/MAPK pathway ASEs were also strikingly enriched in HGG. *FGFR1, NTRK3*, *NF1*, and *BRAF* all had highly ranked ASEs involving key structural features which would be predicted to affect RAS/MAPK activation (Fig. 4a–d and Supplementary Fig. S5). We validated the *FGFR1* and *NF1* ASEs in RNA-Seq from a small independent pHGG cohort (pHGG (*n* = 9), normal brain (*n* = 3); Supplementary Fig. S5g, h)^23^ as well as by RT-PCR (Supplementary Fig. S2d).

*BRAF* splicing in the RAS-binding domain (Supplementary Fig. S5a, b) was predicted to remove BRAF-S151, which is subject to feedback through phosphorylation by ERK to limit RAS/MAPK activation^24^. *NTRK3* splicing is predicted to remove the Y516 autophosphorylation site that is important for RAS activation (Supplementary Fig. S5c, d)^25^. Thus, *BRAF* and *NTRK3* splicing would be expected to increase and decrease MAPK activation, respectively; however, their differential expression in pHGG would oppose this, making interpretation of their functional relevance more challenging (Supplementary Fig. S5b, d). *FGFR1* is expressed in two isoforms. FGFR1-β skips the N-terminal Ig-like domain, has a higher affinity for FGF than FGFR1-α leading to increased downstream signaling and can be skipped in GBM^26,27^. It was also skipped in pHGG, accompanied by *FGFR1* upregulation (Supplementary Fig. S5e, f).

*NF1* exon23a was among the most significant ASE in pHGG (mean ψ increase 46%, *P* < 10^−15^; Fig. 4b–d). This exon is skipped in *NF1-I*, while its inclusion in *NF1-II* inserts a 21-aa loop into the RAS-GTP-binding site, reducing the affinity of NF1 for RAS-GTP^28,29^. *NF1-II* is the predominant non-brain isoform, while *NF1-I* is expressed in the brain (*P* < 10^−15^; Supplementary Fig. S5i). Importantly, bulk expression of *NF1* is unchanged in pHGG and does not correlate with exon23a inclusion (Supplementary Fig. S5j, k). Finally, we further confirmed the differential splicing of *NF1* exon23a using qRT-PCR in three matched pHGG-normal pairs using isoform-specific primers (Supplementary Fig. S5l). Interestingly, we also observed the same isoform switch in three mouse pHGG models^23,30,31^ indicating this is a conserved feature between species (Supplementary Fig. S5m).

The *NF1-II* isoform switch was also evident in adult GBM and IDH-mutant diffuse gliomas (mean ψ increase 50–62%; *P* = 0.01 [junction], *P* ≤ 0.05 [isoform] Fig. 4e and Supplementary Fig. S6a). The isoform switch did not correlate strongly with *NF1* expression, although *NF1* was modestly down- and upregulated in IDH-wt and IDH-mut/codel diffuse glioma, respectively (Supplementary Fig. S6b–e).

**Fig. 5:**
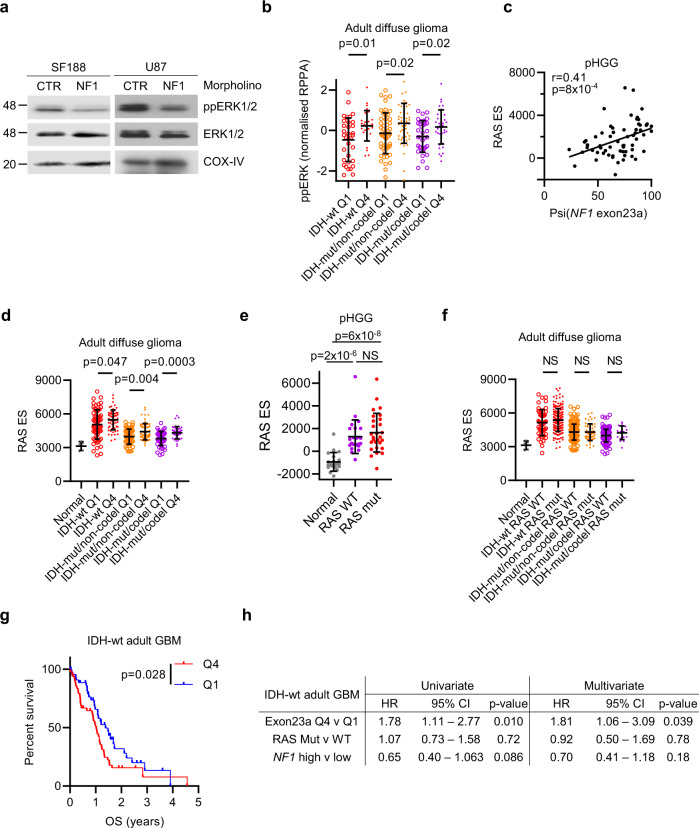
Activation of RAS/MAPK by *NF1* exon23a inclusion in HGG. **a** Western blot of cells harvested 48 h post transfection with control or *NF1* exon23a-specific morpholinos. Gels are representative of two experiments. **b** Normalised reverse-phase protein array (RPPA) signal for phospho-ERK (ppERK) for adult diffuse glioma Q1 and Q4 patients in each subgroup. n: IDH-wt Q1 = 34, Q4 = 30; IDH-mut/non-codel Q1 = 50, Q4 = 51; IDH-mut/codel Q1 = 34, Q4 = 37. Bars show mean ± standard deviation. **c** Plot of *NF1* exon23a inclusion (psi) and RAS pathway activation score (RAS ES; enrichment score, determined from single-sample GSEA [ssGSEA]) in pHGG. r: Pearson correlation. **d** RAS pathway activation ssGSEA score for the normal brain (*n* = 5) and adult diffuse glioma patients that are in quartile 1 (Q1) and quartile 4 (Q4) of *NF1* exon23a inclusion for each subgroup. n: IDH-wt Q1 = 60, Q4 = 58; IDH-mut/non-codel Q1 = 63, Q4 = 63; IDH-mut/codel Q1 = 45, Q4 = 43. Bars show mean ± standard deviation. **e** RAS pathway activation ssGSEA score in normal brain (*n* = 20) or pHGG that is RAS/MAPK WT (*n* = 23) or mutant (*n* = 28). Bars show mean ± standard deviation. **f** RAS pathway activation ssGSEA score for adult diffuse glioma subgroups that are RAS/MAPK WT or mutant. n: IDH-wt WT = 27, mut = 216; IDH-mut/non-codel WT = 165, mut = 105; IDH-mut/codel WT = 165, mut = 105. Bars show mean ± standard deviation. **g** Kaplan–Meier survival plot of (NF1-WT) IDH-wt GBM patients that are in the top (Q4, *n* = 50) or bottom (Q1, *n* = 47) quartile of *NF1* exon23a inclusion. **h** Univariate and multivariate Cox proportional-hazards survival analysis of adult IDH-wt GBM accounting for *NF1* exon23a inclusion (Q4 vs Q1), RAS/MAPK pathway mutation, and *NF1* expression. HR hazard ratio, CI confidence interval. Statistical tests: *t* (**b**, **c**, **d**, **f**), Brown–Forsythe ANOVA with Dunnett’s T3 multiple comparisons test (**e**), Gehan–Breslow–Wilcoxon (**g**), Cox proportional-hazards (**h**). NS not significant. Source data are provided as a Source Data file.

NF1-II has been reported to decrease RAS-GTP turnover, boosting downstream MAPK activity^32,33^. To test the functional consequence of this switch in pHGG and GBM, we blocked *NF1* exon23a inclusion with morpholinos targeting the 3’ and 5’ intron–exon junctions to interfere with spliceosome access. These *NF1* exon23a-specific morpholinos promoted exon23a skipping and increased MAPK signaling in both pediatric and adult HGG cells (Fig. 5a and Supplementary Fig. S7a). Accordingly, increased *NF1* exon23a-high adult diffuse glioma patients (Q4) have significantly increased phospho-ERK (*P* ≤ 0.02; Fig. 5b). Furthermore, gene sets transcriptionally regulated by RAS or MAPK, which are overall highly upregulated in both pHGG and adult diffuse glioma (Fig. S7b–d), are significantly upregulated in tumors with increased *NF1* exon23a inclusion in a manner not related to bulk *NF1* expression (Fig. 5c, d and Supplementary Fig. S7e–g). In contrast, there was no increased RAS/MAPK activation associated with RAS/MAPK mutant pHGG or adult diffuse glioma (Fig. 5e, f, Supplementary Fig. S7h, and Supplementary Data 2).

The functional RAS/MAPK activation by *NF1* isoform switching prompted us to ask whether it was associated with OS. After excluding *NF1*-mutant patients, increased *NF1* exon23a inclusion was associated with worse OS in adult diffuse glioma (*P* = 2 × 10^−5^; Supplementary Fig. S7i). This was specific to IDH-wt GBM (*P* = 0.028) and was independent of *NF1* expression and RAS/MAPK pathway gene mutation on multivariate analysis (*P* = 0.039) (Fig. 5g, h and Supplementary Fig. S7j, k).

Recent work has shown that *NF1* exon23a splicing is regulated by neuronal differentiation and that altering the inclusion rate affects differentiation kinetics^34^, raising the prospect that *NF1* exon23a splicing may also play a role in the differentiation state of HGG. We used an established model of neuronal differentiation of GBM^35^, in which culturing cells in the presence of sodium butyrate in the absence of serum induces differentiation (Supplementary Fig. S8a). We transfected cells with control or NF1-specific morpholinos for 24 h to establish a specific splicing pattern, followed by 48 h of differentiation. In this system, U87 GBM cells exhibited robust induction of differentiation markers compared to untreated cells (Supplementary Fig. S8b). In contrast to neuronal differentiation of PC12 cells where *NF1* exon23a inclusion decreases^34^, *NF1* exon23a inclusion did not change in differentiated U87 cells (Supplementary Fig. S8c).

In differentiating PC12 cells, modulating *NF1* exon23a inclusion affected the dynamics of neuronal differentiation^34^. U87 cells transfected with *NF1*-specific morpholinos to block the inclusion of exon23a did not change the expression of neuronal marker genes in either media condition compared to cells transfected with control morpholino, suggesting that *NF1* exon23a inclusion does not affect HGG cell differentiation in this system and conditions (Supplementary Fig. S8b).

Collectively, these data show that oncogenic pathways can be functionally activated through non-mutagenic processes, such as AS, in HGG, and suggest that mutation profiling alone may be insufficient to identify patients for targeted therapies.

### Alternative splicing of *NF1* exon23a in pHGG is regulated downstream from REST

**Fig. 6:**
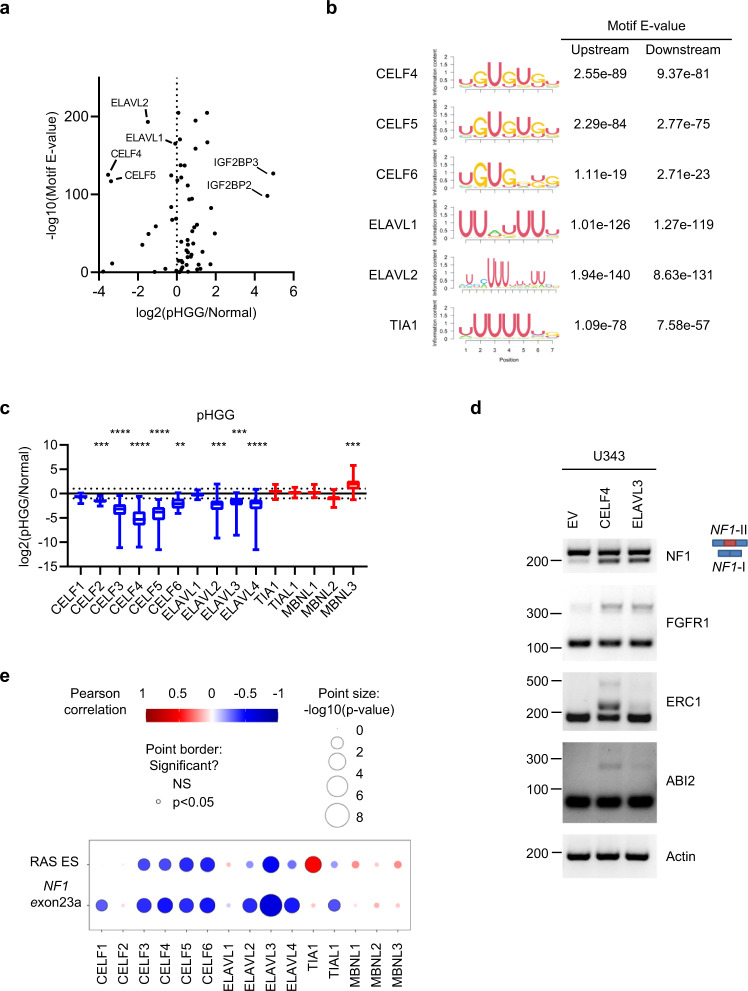
*NF1* exon23a splicing in HGG is controlled by differential expression of the CELF/ELAVL gene families. **a** Scatter plot comparing log2 fold change in expression between pHGG and normal brain of splice factors and RNA-binding proteins, with the enrichment of the RNA motifs they bind in introns surrounding differentially spliced exons in pHGG. **b** Motifs and enrichment in up- and downstream introns surrounding differentially spliced exons for the indicated splicing factors. **c** Expression ratio (log2 fold change) of negative (blue) and positive (red) NF1 exon23a splicing regulators between pHGG (*n* = 64) and normal brain (*n* = 20). Box shows IQR with the median marked, and the whiskers show the range. *P* values: CELF2, 0.0001; CELF3, 4 × 10^−16^; CELF4, 7 × 10^−28^; CELF5, 2 × 10^−26^; CELF6, 0.001; ELAVL2, 0.0003; ELAVL3, 0.0001; ELAVL4, 1.5 × 10^−5^; MBNL2, 0.01, MBNL3, 7 × 10^−5^. **d** U343 GBM cells transduced with control (EV) lentivirus or expressing CELF4 or ELAVL3 were analyzed by RT-PCR for the indicated splicing events. Bands corresponding to *NF1*-II (includes exon23a, red box) and *NF1*-I are shown at the left. Gels are representative of two experiments. **e** Bubble plot showing the relationship between *NF1* exon23a splicing regulators and *NF1* exon23a inclusion and RAS activity score (ES, enrichment score from ssGSEA) in pHGG. Bubble color represents Pearson correlation, size is significant, and statistically significant correlations have a black border on the bubble. Statistical tests: *t* (**c**, **e**). *P < 0.05, **P < 0.01, ***P < 0.001. Source data are provided as a Source Data file.

As a family, splicing factors are widely differentially expressed in pHGG (Fig. 1b). We compared the differential expression of RNA-binding proteins including splice factors with enrichment of their binding motifs in the introns up- and downstream from differentially spliced exons, finding that members of the CELF and ELAVL families are the most downregulated genes with enriched motifs (Fig. 6a). IGF2BP2/3, which is known to promote tumorigenic behavior through stabilization of *HMGA* mRNA and is widely overexpressed in cancer^36^, is strongly upregulated in pHGG with accompanying enrichment of its binding motif (Fig. 6a).

The CELF/ELAVL enrichment was intriguing given that the CELF/ELAVL families have been reported to repress *NF1* exon23a inclusion while the MBNL and TIA1/TIAL1 families promote it^37,38^ (Supplementary Fig. S9a). Consistent with intronic CELF/ELAVL binding sites surrounding exon23a, their motifs are enriched both up- and downstream of differentially spliced exons in pHGG, respectively (Fig. 6b and Supplementary Fig. S9a). Furthermore, the *CELF*/*ELAVL* families, in particular *CELF3-5* and *ELAVL2-4*, are downregulated in pHGG while TIA/TIAL1 and MBNL3 are modestly upregulated (Fig. 6c and Supplementary Fig. S9b). The same pattern was found in adult diffuse glioma, albeit less pronounced in IDH-mut/codel oligodendroglioma (Supplementary Fig. S9c–e).

We hypothesized that family-wide *CELF*/*ELAVL* downregulation could explain the increased *NF1* exon23a inclusion we observed in HGG. By expressing CELF4 and ELAVL3 in U343 GBM and HEK293T cells, we confirmed by RT-PCR that they suppress inclusion of *NF1* exon23a (Fig. 6d and Supplementary Fig. S6f). The enrichment of CELF/ELAVL motifs surrounding differentially spliced exons imply they regulate many more differential ASE in pHGG. To assess this possibility, we identified genes with binding sites surrounding differential exons. By RT-PCR we could validate select additional targets beyond *NF1*, including the FGFR1 ASE (Fig. 6d). Importantly, for each event we tested, the expression of CELF4/ELAVL3 modulated its splicing in the opposite direction to that induced in pHGG, supporting the idea that these families regulate the pHGG AS landscape.

Increased inclusion of *NF1* exon23a in HGG leads to increased RAS/MAPK activation (Fig. 5 and Supplementary Fig. S7). We therefore next compared the expression of *CELF*/*ELAVL* genes in pHGG with RAS activity as well as *NF1* exon23a inclusion, finding that these families (especially *CELF3-6* and *ELAVL2-4*) are significantly negatively correlated with both *NF1* exon23a inclusion and RAS activity (Fig. 6e).

**Fig. 7:**
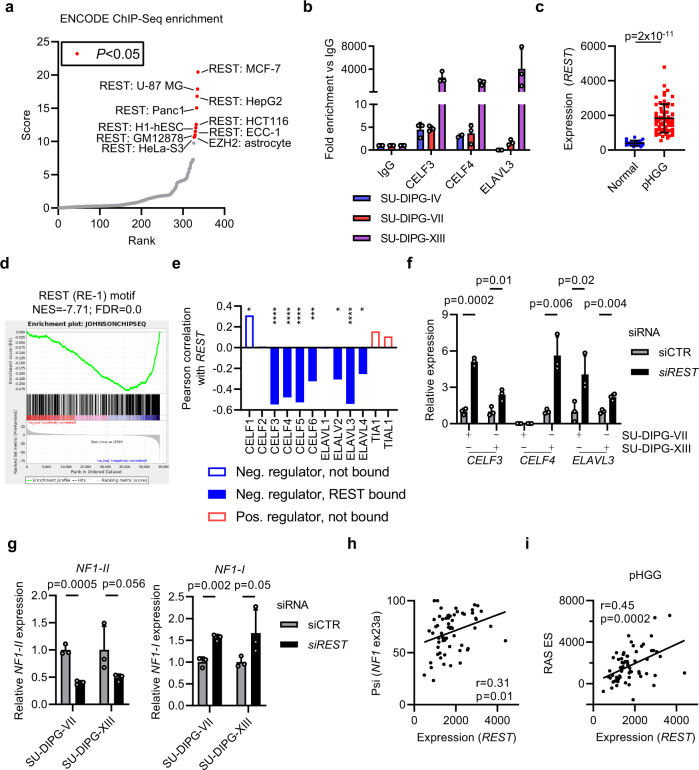
NF1 exon23a splicing in pHGG is regulated downstream from REST. **a** Enrichment of transcription factor binding sites in *NF1* exon23a splice regulators were calculated by Enrichr using ENCODE ChIP-Seq data. All significant results (FDR < 0.05, red) are highlighted red and the dataset labeled. **b** ChIP was carried out using REST or IgG antibodies with chromatin from primary DIPG cell lines and analyzed as percent input at the indicated loci (*n* = 3). Bars show mean ± standard deviation. **c** REST expression in pHGG (*n* = 64) and normal brain (*n* = 20). Bars show mean ± standard deviation. **d** Gene set enrichment analysis of REST signature genes with a REST-bound RE1 motif. NES normalized enrichment score, FDR false discovery rate. **e** Pearson correlation of REST expression and expression of NF1 exon23a splice regulators (CELF1, *P* = 0.01; CELF3; *P* = 10^−5^, CELF4, *P* = 10^−4^, CELF5, *P* = 10^−6^, CELF6, *P* = 0.01; ELAVL2, *P* = 0.01; ELAVL3, *P* = 10^−5^, ELAVL4, *P* = 0.04). Genes bound by REST across ENCODE ChIP-Seq datasets are shaded in solid color, genes not bound by REST are empty. **f** qRT-PCR in SU-DIPG-VII and SU-DIPG-XIII cell lines transfected with control or *REST*-specific siRNA. Bars show mean ± standard deviation (*n* = 3) relative to 18S housekeeping, normalized to siCTR expression for each gene. **g** qRT-PCR in SU-DIPG-VII and SU-DIPG-XIII cell lines transfected with control or *REST*-specific siRNA. Bars show mean ± standard deviation (*n* = 3) relative to 18S housekeeping, normalized to siCTR expression for each gene. **h** Scatter plot of *REST* expression and *NF1* exon23a inclusion in pHGG. r: Pearson correlation. **i** Scatter plot of *REST* expression and RAS pathway activity (ssGSEA ES) in pHGG. r: Pearson correlation. Statistical tests: *t* (all; multiple *t* tests with two-stage linear step-up procedure of Benjamini, Krieger, and Yekutieli (**f**, **g**)). *P < 0.05, **P < 0.01, ***P < 0.001, ****P < 0.0001. Source data are provided as a Source Data file.

In all, 14% (*n* = 13/90) of pHGG had an SNV (*n* = 9) or CNV (*n* = 4) in a *CELF*/*ELAVL* family gene, suggesting that both mutagenic and non-mutagenic processes are responsible for this mechanism. To identify potential regulators of these genes, we profiled ENCODE ChIP-Seq data to find transcription factors binding their promoters. The significant hits across all cell lines were near-exclusively the RE1 silencing transcription factor (REST; Fig. 7a), which has recently been shown to be important for pHGG and GBM cell growth amongst other cancers^39–42^. Strong REST peaks^43^ were observed in GBM cells associated with *NF1* exon23a splice regulators, which we confirmed for the same sites in *CELF3*, *CELF4,* and *ELAVL3* in three primary pHGG cell lines by ChIP-qPCR (Fig. 7b and Supplementary Fig. S9g).

*REST* was significantly upregulated in pHGG (*P* < 0.0001) and, as expected for a repressive transcription factor, expression of RE1 motif-containing, REST-bound genes was highly downregulated (Fig. 7c, d)^44^. In particular, *REST* directly binds most CELF/ELAV and is negatively associated with their repression (Fig. 7e), suggesting that it binds and represses their expression in pHGG. To test this hypothesis, we depleted *REST* from primary pHGG cells, finding significant increases in *CELF3*, *CELF4*, and *ELAVL3* expression (Fig. 7f and Supplementary Fig. S9h). In turn, *REST* depletion caused a significant increase in *NF1-I* expression with an accompanying decrease in *NF1-II* (Fig. 7g). Consistent with this, increased *REST* expression was significantly associated with *NF1* exon23a splicing and RAS/MAPK activation (*P* ≤ 0.01; Fig. 7h, i and Supplementary Fig. S9i). Together, this supports a mechanism by which *REST* upregulation in pHGG drives increased RAS/MAPK signaling by promoting *NF1* exon23a inclusion (Supplementary Fig. S9j).

## Discussion

Here, we found that pHGG has increased alternative splicing (AS) burden compared with the normal brain. In some tumors, this is associated with spliceosome mutations. Previous work identified sporadic AS alterations in adult cancers, as well as mutations or expression changes in splicing factors and direct mutations to splice sites within genes^10,45,46^. We found the earliest assembly (complex E) and assembled catalytic (Bact) stages of the spliceosome to be particularly affected, suggesting that target site recognition and initiation of catalysis are the key determinants driving this pattern. Given the lethality of spliceosome inhibitors towards pHGG/GBM lines it is likely that the spliceosome mutations acquired in pHGG do not render the spliceosome completely inactive but rather alter the activity of the spliceosome. In addition to mutations in the spliceosome itself affecting AS in pHGG, not every case had mutations in the spliceosome suggesting other mechanisms are also at play, including differential expression of spliceosome components/splicing factors that could be regulated by transcriptional or epigenetic rewiring. Furthermore, the widespread epigenome remodeling that takes place in HGG suggests that this could also have an impact on AS given the known effects of histone and DNA methylation on splicing^47^.

Tumors each acquire mutations in a small subset of cancer drivers^3,9,20^. We found their burden of AS changes to be significantly higher, not only in pHGG but also in a broad range of adult cancers, implying that AS is an under-appreciated mechanism of oncogenic pathway activation. In adult diffuse glioma, which exhibited the biggest differences between cancer-driver mutation and AS burden, the latter also conferred a worse prognosis.

Differential ASEs in pHGG converged on cancer-driver genes, particularly the RAS/MAPK and chromatin modification pathways, and were predicted to have functional consequences. We showed that the *NF1* isoform switch, which has previously been identified in RT-PCR analyses of adult GBM^48,49^, is driven by REST-mediated suppression of exon23a splice regulators and leads to activation of the RAS/MAPK pathway. Significantly, the NF1 isoform switch was more closely associated with signatures of RAS/MAPK activation than mutations in RAS/MAPK pathway member genes. Additional ASE such as that found in *FGFR1* would also be expected to lead to elevated RAS/MAPK signaling.

Germline alterations in *NF1* drive neurofibromatosis type I, which predisposes patients to develop a range of nervous system tumors including neurofibroma and glioma^50^. Although *NF1* is mutated in around 10% of both pediatric and adult HGG, our findings demonstrate a near-universal mechanism of RAS/MAPK activation through AS-mediated *NF1* inactivation. Signaling downstream from activated growth-factor receptors can be attenuated through a variety of feedback mechanisms, including activation of NF1 by the ribosomal S6 kinase (RSK)^51^. Receptors are the main RAS/MAPK pathway mutation targets in HGG^3,4^. As well as reducing activity toward RAS, switching isoforms to NF1-II in HGG will therefore reduce the ability of tumor cells to dampen their response to constitutive pathway activation as well as ligand-mediated signaling through other receptors.

In summary, our data demonstrate that tumors exploit non-mutagenic methods to activate oncogenic processes and, more broadly, that the absence of a mutation does not mean that oncogenic pathways will be inactive, just as the presence of a mutation in a pathway does not guarantee its activity. The decision to treat a patient with a particular targeted agent should not necessarily be based solely on mutational profiling but should also incorporate other molecular information such as splicing alterations and measures of pathway activation.

## Methods

### Ethical approval and patient samples

Work involving patient material was approved by the Hospital for Sick Children Research Ethics Board (#1000055059). Written consent from a legally authorized representative (all patients in this study were under 18) was obtained to collect tissue for research in all autopsy cases and in all surgical cases collected since 2010, with explicit consent for use for next-generation sequencing since 2016. For surgical cases prior to 2010 or where the consent did not explicitly state the tissue would be used for next-generation sequencing, for deceased patients, waiver of consent to use the tissue for this purpose was granted by the Hospital for Sick Children Research Ethics Board.

### Reagents and plasmids

Morpholinos were from GeneTools (Oregon, USA), targeted against the *NF1* intron30/exon31(exon23a) and exon31(exon23a)/intron31 junctions or a pre-designed non-targeting control. Sequences were analyzed by BLAST to confirm there were no high-specificity off-target predicted binding sites. Sequences are listed in Supplementary Table 1.

*CELF4* and *ELAVL3* were amplified from cDNA generated from 293T cells and cloned between the XbaI/BamHI sites of a pCDH-CMV-MCS-EF1α-copGFP (SystemBioscience) modified to encode a FLAG/HA tag between the BamHI/NotI sites. Primer sequences are listed in Supplementary Table 2. All plasmids were sequenced before use.

### Cell culture and lentivirus generation

SF188, U87, U343, fetal NHA, and HEK293T cell lines were maintained in DMEM (VWR) supplemented with 10% FBS (Wisent) and 1% PenStrep (Invitrogen). SF188 cells (RRID CVCL_6948) were a kind gift from Chris Jones (Institute for Cancer Research, London). U87 (RRID CVCL_0022), U343 (RRID CVCL_4773), and HEK293T (RRID CVCL_0045) cells were from ATCC. Fetal NHA cells (T0281) were from ABM. SU-DIPG-IV (RRID CVCL_IT39), SU-DIPG-VI (RRID CVCL_IT40), SU-DIPG-VII, SU-DIPG-XIII (RRID CVCL_IT41), SU-DIPG-XVII, and SU-DIPG-XXXVI were a kind gift from Michele Monje (Stanford University)^52^ and maintained in Neurobasal(−A) media (Invitrogen) supplemented with B27(-A; Fisher-Gibco), growth factors (human-bFGF [20 ng/ml], human-EGF (20 ng/ml), human PDGF-AA (20 ng/ml), human PDGF-BB (20 ng/ml); Gemini Bio-products) and heparin (10 ng/ml; Fisher/Stem Cell Technologies), as previously described^52^.

Plasmids were packaged into lentivirus by cotransfection into HEK293T cells with psPAX2 (Addgene#12260) and pMD2.G (Addgene#12259) using Lipofectamine 2000 (Invitrogen). The media was changed the following day and the supernatant was collected after 30 h and precipitated overnight with Lenti-X concentrator (Clontech) before being resuspended in Optimem (Invitrogen). Cells were transduced for 24 h in the presence of 10 μl/ml polybrene (Santa Cruz).

Cell viability assays were carried out by seeding cells in 96-well or six-well plates. The next day pladienolide B, madrasin (Cayman chemicals) or DMSO control was added and cells were incubated for 4 days. alamarBlue (Invitrogen) was added for 4 h and measured in a fluorescence plate reader (Molecular Devices) by exciting at 560 nm and measuring emission at 590 nm, or cells were stained with trypan blue and counted. Viability was determined relative to DMSO control.

Morpholino transfections were carried out using Endo-Porter reagent (GeneTools) according to the manufacturers’ instructions. Both NF1 morpholinos were simultaneously transfected each at a final concentration of 1 µM (control morpholino was used at 2 µM), which allowed specific modulation of exon23a splicing without affecting *NF1* expression. Cells were harvested after 48 h or, for differentiation experiments, 72 h.

Differentiation assays were carried out as described^35^. In all, 200,000 U87 cells were seeded in six-well plates in standard media and transfected the next day with control or *NF1*-specific morpholinos. After 24 h, media was changed either with standard media (control) or differentiation media (DMEM without serum, supplemented with 4 mM sodium butyrate).

For siRNA transfections, 50,000 SU-DIPG-IV or SU-DIPG-VII cells were seeded in 24-well plates supplemented as above and transfected with either human REST smartpool or Non-targeting plus control siRNA (Dharmacon) at a final concentration of 20 nM with DharmaFECT 1 reagent (Dharmacon) according to the manufacturers’ instructions. Cells were harvested after 48 h.

### RNA extraction and analysis

Total RNA was extracted from cells or fresh-frozen tissue samples using the RNeasy kit (QIAGEN). Total RNA was reverse transcribed with 5× All-In-One RT MasterMix (Applied Biological Materials). Samples were analyzed either by reverse transcriptase PCR (RT-PCR) followed by visualization on a 2% agarose gel, or quantitative RT-PCR (qRT-PCR) with iTaq Universal SYBR green supermix (Bio-Rad) using a StepOnePlus machine (Applied Biosystems). Alternately, RNA was extracted from cells the the Quick-RNA MiniPrep Kit (Zymo Research) and cDNA synthesized with the iScript cDNA Synthesis Kit (Bio-Rad). Samples were analyzed by qRT-PCR with a 2× SensiMix SYBR & Fluorescein Kit (Bioline) on a Lightcycler 96 machine (Roche).

Expression from qRT-PCR was normalized using the delta-delta Ct using either beta-actin or 18S housekeeping genes. Primers sequences are listed in Supplementary Table 1.

### Western blotting

Extracts from cells lysed in 2x SDS lysis buffer (20 mM Tris (pH 7.4), 20 mM EDTA, 2% SDS, 20% glycerol) were resolved using 10-20% SDS-PAGE gels (Invitrogen) and transferred to PVDF membranes (Amersham). Membranes were blocked and incubated overnight with primary antibody diluted in 3% BSA in TBS with 0.1% Tween-20. After incubation with HRP-conjugated secondary antibodies (Jackson), the signal was detected with enhanced chemiluminescence (Pierce). Primary antibodies used were from Cell Signaling: ERK1/2 (9102, 1:1000), phospho-ERK1/2 (Thr202/204; 9101, 1:1000), COX-IV (clone 3E11; 4850, 1:1000).

### Chromatin immunoprecipitation

Actively dividing SU-DIPG (IV, VII, and XIII) cells were fixed with 1% formaldehyde. Cell pellets (5 million per reaction) were suspended in lysis buffer (50 mM Tris-HCl (pH 8.0), 10 mM EDTA (pH 8.0), 1% SDS, protease inhibitors (Sigma) and chromatin sonicated with a Bioruptor Pico (Diagenode). 1% of the recovered material was saved as input and the remainder diluted fivefold in ChIP dilution buffer (16.7 mM Tris-HCl (pH 8.0), 167 mM NaCl, 1.2 mM EDTA (pH 8.0), 1.1% Triton X-100, protease inhibitors) and precleared. REST (Millipore, 07-579) or IgG (Santa Cruz, sc2027) antibodies were added overnight before incubation with protein-A beads (Millipore) and washing once each with low-salt, high-salt, and lithium chloride immune complex buffers, and twice with TE buffer. DNA was eluted with 1% SDS and 0.1 M NaHCO_3_ and cross-links reversed by incubation with NaCl for 4 h at 65°. DNA was purified with PCR purification kit (Zymo Research) and analyzed by qPCR with a 2× SensiMix SYBR & Fluorescein Kit (Bioline) on a Lightcycler 96 machine (Roche).

### Whole-exome/genome sequencing analysis

DNA was extracted from fresh-frozen tissue samples with DNeasy kit (QIAGEN). Exome libraries were generated and sequenced at The Centre for Applied Genomics, Hospital for Sick Children. Libraries were either Ion TargetSeq Exome 50 Mb (ThermoFisher, sequenced on Ion Proton machines and aligned to human genome hg19 with Torrent Suite Software) or TruSeq Exome (Illumina, sequenced on HiSeq 2500 machines). TruSeq exome reads were trimmed with Trimmomatic-v0.32^53^, aligned with bwa-mem-v0.7.8^54^, processed with the GATK suite^55^ and duplicate reads were marked with Picard-v2.5.0 (http://broadinstitute.github.io/picard). Variants were called with VarScan-v2.3.6^56^ and annotated with SnpEff-v4.3k^57^. To verify variants, a second algorithm was used, either GATK HaplotypeCaller for Illumina sequencing or Torrent Unified Variant Caller (ThermoFisher) for Ion Proton sequencing and key variants were further manually verified using the IGV browser. Copy number alterations were called with CNVkit-v0.8.6^58^.

Samples were categorized into mutant groups according to the presence or absence of an alteration in a core gene of the RAS/MAPK pathway (Supplementary Data 2), the major spliceosome (HUGO group 1518; Supplementary Data 2), or the COSMIC cancer census. To be counted as mutant, either an SNV/indel with medium/high impact (missense, frameshift, nonsense, and splice site mutations) at variant-allele frequency >0.2 and depth >20, a copy number gain with 5+ copies, or a homozygous deletion had to be present in the sample for any pathway gene. To be counted as wild-type, all genes in the pathway were required to be free of both SNV/indels and copy number changes. The same criteria were used for spliceosome mutations.

Where we had previously analyzed samples by WGS, WES, and SNP6.0 array to identify mutations and copy number alterations (Supplementary Data 1)^2^, we used these data to infer the mutation status of the RAS pathway and spliceosome.

Mutation data for TCGA^4,7,59^ and CBTTC^11,12^ datasets were downloaded from cBioPortal and PedcBioPortal, respectively. TCGA SNV calls were generated with Mutect and IndelLocator^4,7,59^, and CBTTC SNV calls come from Strelka^11,12^.

### RNA sequencing analysis

The quality of total RNA isolated from fresh-frozen tissue was confirmed with a Bioanalyzer 2100 (Agilent). Libraries were prepared with TruSeq Stranded Total RNA Library Prep with Ribo-Zero Gold kits (Illumina, CA, USA) and sequenced at The Hospital for Sick Children with 100 bp or 125 bp paired-end reads on Illumina HiSeq 2500 instruments. Data were processed with the GenPipes framework^60^. Reads were quality trimmed with a maximum length of 100 bp with Trimmomatic-v0.32^53^ and aligned to human genome build version GRCh37-v75 using STAR-v2.5.0 in 2-pass mode^61^. Duplicate reads were marked with Picard-v2.5.0.

Differential expression analysis was carried out by counting expression with HTSeq^62^ and testing with edgeR and DESeq^63,64^; only those genes with absolute fold change >2 and a Benjamini–Hochberg adjusted *P* value <0.05 called by both edgeR and DESeq were considered to be differentially expressed. Pre-ranked gene set enrichment analysis (GSEA)^65^ was carried out by ranking genes with the product of their fold-change sign and the -log10(adjusted *P* value). t-SNE analyses were carried out with the Rtsne package in R. The RAS expression signature is from the MSigDB Hallmarks signature set and the MAPK signature from PROGENy^66,67^.

Transcript quantification and single-sample GSEA (ssGSEA) was carried out by aligning the trimmed reads to the transcriptome using RSEM-v1.2^68^. ssGSEA was carried out using the GenePattern server after discarding genes with mean FPKM < 1.

Splicing patterns across samples were quantified using rMATS-v4.0.1^16^ and SUPPA2^18^. Sashimi plots were drawn with rmats2sashimiplot (https://github.com/Xinglab/rmats2sashimiplot). Events with inclusion change >15% and FDR < 0.05 were considered to be significant. Motif enrichment was determined with the AME tool from MEME Suite-v5.4.1 using CIS-BP RNA motifs^69,70^. Enriched Reactome pathways were determined in Cytoscape-v3.8.2^71^ using ClueGO-v2.5.7^72^ with Bonferroni-adjusted *P* < 0.05 and clustered according to similarity. Protein–protein interactions with experimental or database evidence were retrieved from the STRING database^73^ and visualized in Cytoscape. Pre-ranked GSEA for splicing events was carried out by ranking genes based on the -log10(adjusted *P* value) for the most significant event for each gene.

The amount of splicing was calculated using RSEM transcript quantification for genes with multiple transcripts. Using the R ‘entropy’ package, Shannon entropy $H$ for a gene $G$ with $g$ isoforms was calculated as:

1 $$H(g)=-\mathop{\sum}\limits_{i=1}^{g}{P}_{i}.\,\log ({P}_{i})$$

where ${P}_{i}$ is the probability of each transcript being expressed based on transcript ratios. The median of $H$ for groups of samples was analyzed. The variability of ASE inclusion in pHGG and normal brain was assessed by plotting cumulative distribution functions of the standard deviation of ASE.

Splicing burden in COSMIC driver genes for pHGG and TCGA data were determined by identifying samples with maximal ASE inclusion change >30% compared with normal tissue for each gene in the census.

Mouse RNA-Seq data were processed as above, except that mouse genome build version GRCm38-v83 was used.

### Statistical analysis

Kaplan–Meier curves were drawn and Gehan–Breslow–Wilcoxon tests were carried out using GraphPad Prism 8. Unless otherwise stated, all tests are two-tailed Student’s *t* test, not assuming equal variance between samples, and were carried out in GraphPad Prism 8 or R-3.6.1.

### Reporting summary

Further information on research design is available in the Nature Research Reporting Summary linked to this article.

## Supplementary information

Supplementary Information

Description of Additional Supplementary Files

Supplementary Data 1

Supplementary Data 2

Supplementary Data 3

Supplementary Data 4

Reporting Summary

## Data Availability

The publicly available total RNA-Seq data from 9 pHGG and 3 normal brains are available from Gene Expression Omnibus (GEO) under accession number GSE95169^23^. The publicly available mouse pHGG model RNA-Seq data are available from GEO under accession numbers GSE120884^30^, GSE95169^23^, and GSE108364^31^. The publicly available REST ChIP-Seq data are available from GEO under accession number GSE32465^43^. TF-binding sites in *CELF*/*ELAVL* gene promoters were identified with Enrichr (https://maayanlab.cloud/Enrichr/)^74^. RE1 motif-containing, REST-bound genes were identified from REST ChIP-Seq analysis^44^. Level 3 processed adult diffuse glioma RNA-Seq (RSEM quantifications of genes and isoforms), RPPA and clinical data were downloaded from The Cancer Genome Atlas/Broad Firehose (https://gdac.broadinstitute.org/), and mutations and copy number variations in the same samples were assessed with cBioPortal (https://www.cbioportal.org/). Only primary tumors were considered^4,7,59^. TCGA RNA-Seq splicing data was retrieved from TCGASpliceSeq (https://bioinformatics.mdanderson.org/TCGASpliceSeq/singlegene.jsp)^75^. CBTTC^11,12^ data were retrieved from PedcBioPortal (https://pedcbioportal.org/). DNA methylation data are available in GEO under accession numbers GSE49822 and GSE55712^21,76^. Probes associated with genes contained in either the KEGG spliceosome or Reactome mRNA splicing pathway ontologies (Fig. 1b) were analyzed. Previously published data on our cohort^2,30^ are deposited in GEO or the European Genomics Archive (EGA); SNP6.0 data are available in GEO under accession number GSE50024; WGS data are available in EGA under accession number EGAS00001000575; WES data are available in EGA under accession numbers EGAS00001000575 and EGAD00001006450; RNA-Seq data are available in EGA under accession number EGAD00001006450. The data newly generated on our cohort is deposited with EGA; WES data are available under accession number EGAD00001008278; RNA-Seq data are available under accession EGAD00001008279. The data are available under controlled access to comply with data protection regulations, and can be accessed by application to the data access committee via C.H. (cynthia.hawkins@sickkids.ca). The remaining data are available within the Article, Supplementary Information, or Source Data file. Source data are provided with this paper.

## Source data

Source Data

## Code availability

Custom code was not used in this study. Parameters used for data processing can be found at https://github.com/rsiddaway/NF1_paper_scripts or https://zenodo.org/record/5735134 and the corresponding DOI is as follows: 10.5281/zenodo.5735134.
