# Supplementary material for: Splicing is an alternate oncogenic pathway activation mechanism in glioma
Source: Nat Commun. 2022 Jan 31;13:588. doi: 10.1038/s41467-022-28253-4 (PMC8803922; doi:10.1038/s41467-022-28253-4)
Supplement: Supplementary file 1 — Supplementary Information [file 41467_2022_28253_MOESM1_ESM.pdf]

Supplementary Information

Siddaway et al., Splicing is an alternate oncogenic pathway activation mechanism in glioma

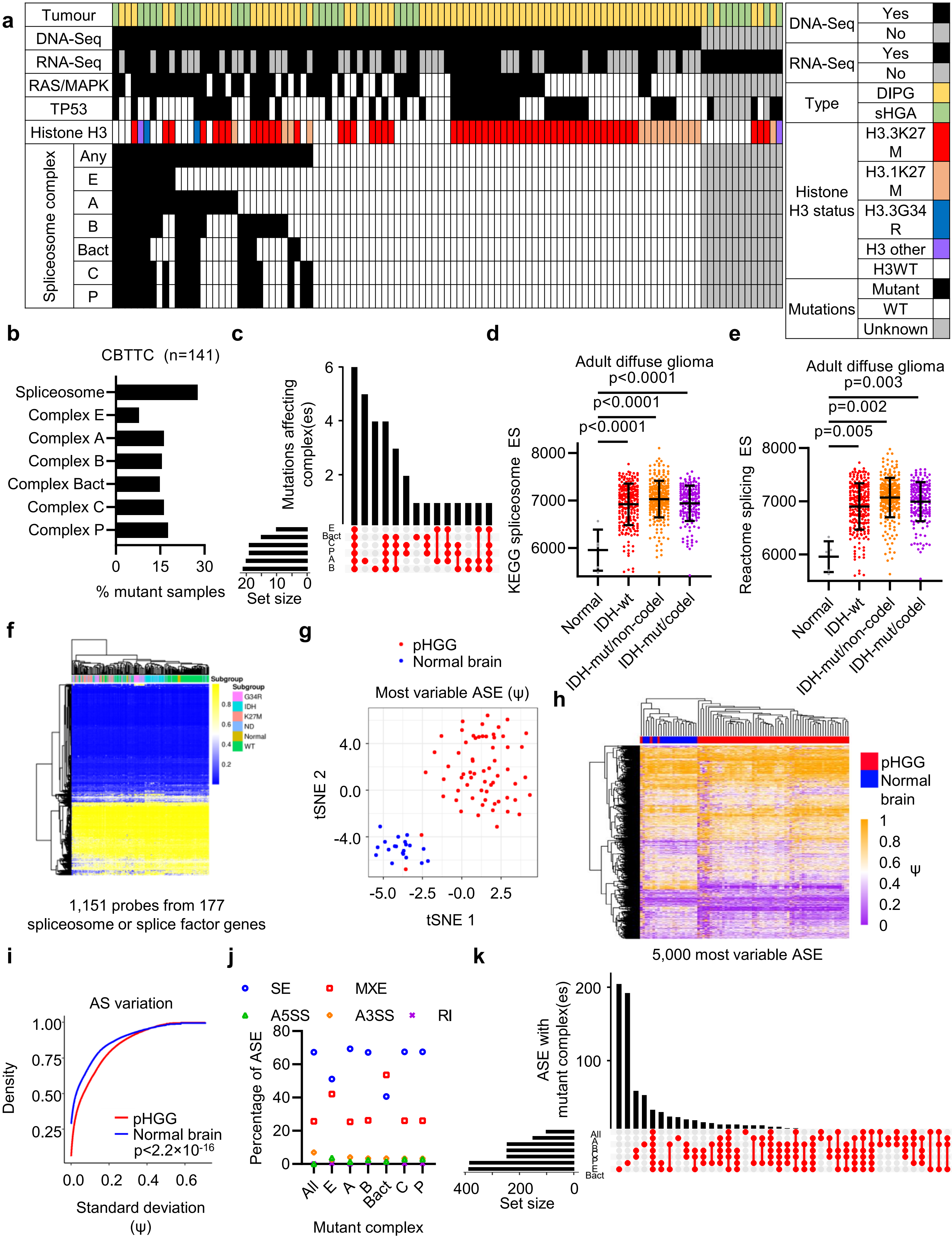

a
Tumour
DNA-Seq
RNA-Seq
RAS/MAPK
TP53
Histone H3
Spliceosome complex
Any
E
A
B
Bact
C
P
DNA-Seq
Yes
No
RNA-Seq
Yes
No
Type
DIPG
sHGA
Histone H3 status
H3.3K27M
H3.1K27M
H3.3G34R
H3 other
H3WT
Mutations
Mutant
WT
Unknown
b
CBTTC (n=141)
Spliceosome
Complex E
Complex A
Complex B
Complex Bact
Complex C
Complex P
0 15 30
% mutant samples
c
Mutations affecting complex(es)
Set size
d
Adult diffuse glioma
p<0.0001
p<0.0001
p<0.0001
KEGG spliceosome ES
Normal
IDH-wt
IDH-mut/non-codel
IDH-mut/codel
e
Adult diffuse glioma
p=0.003
p=0.002
p=0.005
Reactome splicing ES
Normal
IDH-wt
IDH-mut/non-codel
IDH-mut/codel
f
Subgroup
G34R
IDH
K27M
ND
Normal
WT
1,151 probes from 177 spliceosome or splice factor genes
g
pHGG
Normal brain
Most variable ASE (ψ)
tSNE 2
tSNE 1
h
pHGG
Normal brain
ψ
5,000 most variable ASE
i
AS variation
Density
pHGG
Normal brain
p<2.2×10-16
Standard deviation (ψ)
j
SE
MXE
A5SS
A3SS
RI
Percentage of ASE
All
E
A
B
Bact
C
P
Mutant complex
k
ASE with mutant complex(es)
All
A
B
C
P
E
Bact
Set size

Supplementary Figure 1. Spliceosome mutations and alternative splicing in pHGG

A. Oncoprint of samples used in the study showing clinical characteristics as well as common and spliceosome complex mutation status.
B. Frequency of spliceosome mutations in pHGG in total (Spliceosome) and by subcomplex for CBTTC dataset (n=141).
C. UpSet plot showing the distribution of mutations in pHGG across different spliceosome complexes. The top graph shows the number of mutations in genes shared by the combination of complexes at the bottom (red dots connected by lines). The graph at bottom left shows the number of pHGG with mutations affecting a given complex.
D. Single sample gene set enrichment analysis (ssGSEA) of spliceosome component expression in normal brain (n=5) and IDH-wt (n=243), IDH-mut/non-codel (n=270) and IDH-mut/codel (n=173) adult diffuse glioma. Bars show mean ± standard deviation. ES: enrichment score.
E. Splicing pathway single sample gene set enrichment analysis (ssGSEA) of mRNA splicing pathway genes in normal brain (n=5) and IDH-wt (n=243), IDH-mut/non-codel (n=270) and IDH-mut/codel (n=173) adult diffuse glioma. Bars show mean ± standard deviation. ES: enrichment score.
F. Heatmap showing DNA methylation beta-value of probes associated with spliceosome components and spliceosome genes in pHGG (n=230) and normal brain (n=7)[23,77].
G. t-SNE of the 5,000 most variable alternately spliced events (ASE) across pHGG and normal brain.
H. Heatmap of 5,000 most variable ASE between pHGG and normal brain.
I. Cumulative distribution function plots comparing the standard deviation of each ASE in normal brain and pHGG.
J. Percentage of differential ASE ($|\Delta\psi|>0.15$, FDR<0.05) identified between pHGG with mutations in the indicated spliceosome complex and pHGG with WT spliceosomes that come from each ASE category. See also Supplementary Figure 2a for schematic of event types.
K. UpSet plot showing overlaps in the unique ASE identified in spliceosome-mutant pHGG. The top graph shows the number of ASE that were differentially spliced in pHGG with the combination of mutant spliceosome complexes shown at the bottom (red dots). The graph at bottom left shows the number of differential ASE associated with each complex.

Statistical tests: Brown-Forsythe ANOVA with Dunnett's T3 multiple comparisons test (D, E), 2-sided Kolmogorov-Smirnov (H). Source data are provided as a Source Data file.

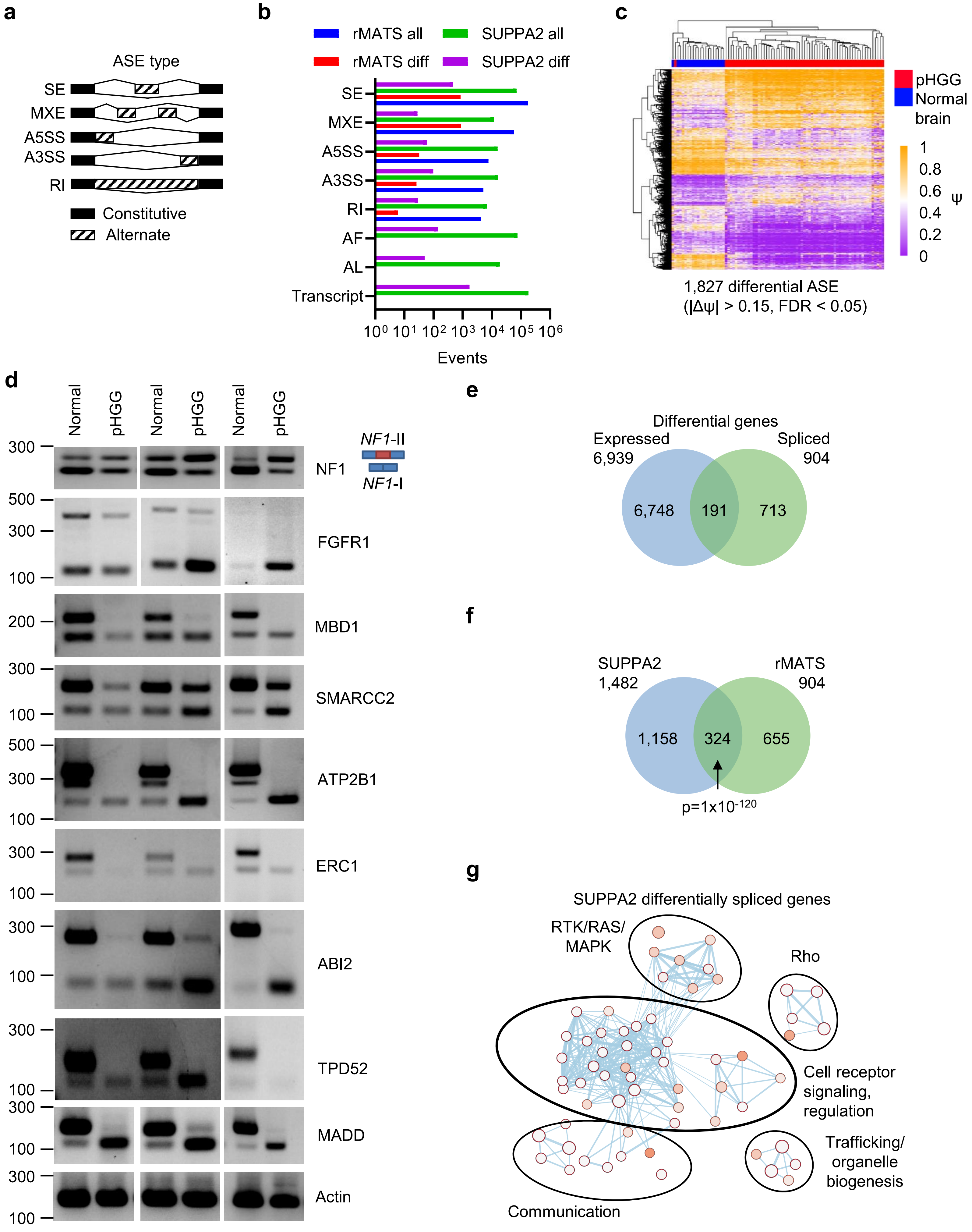

a
ASE type
SE
MXE
A5SS
A3SS
RI
Constitutive
Alternate
b
rMATS all
SUPPA2 all
rMATS diff
SUPPA2 diff
SE
MXE
A5SS
A3SS
RI
AF
AL
Transcript
10^0 10^1 10^2 10^3 10^4 10^5 10^6
Events
c
pHGG
Normal brain
1
0.8
0.6
0.4
0.2
0
ψ
1,827 differential ASE
(|Δψ| > 0.15, FDR < 0.05)
d
Normal
pHGG
Normal
pHGG
Normal
pHGG
NF1
NF1-II
NF1-I
FGFR1
MBD1
SMARCC2
ATP2B1
ERC1
ABI2
TPD52
MADD
Actin
300
500
300
100
200
300
100
500
300
100
300
100
300
100
300
100
300
100
300
100
e
Differential genes
Expressed
6,939
Spliced
904
6,748
191
713
f
SUPPA2
1,482
rMATS
904
1,158
324
655
p=1x10^-120
g
SUPPA2 differentially spliced genes
RTK/RAS/MAPK
Rho
Cell receptor signaling, regulation
Trafficking/organelle biogenesis
Communication

Supplementary Figure 2. AS landscape of pHGG

A. Cartoon of alternative splice events (ASE) characterised. SE: skipped exon. MXE: mutually exclusive exon. A5SS: alternative 5' splice site. A3SS: alternative 3' splice site. RI: retained intron.
B. Number of total and differential splicing events by rMATS and SUPPA2. SUPPA2 additionally characterises splicing events based on alternate first (AF) and last (AL) exons, as well as through switches in isoform abundances.
C. Heatmap of differential alternately spliced events (ASE; $|\Delta\psi|>0.15$, FDR<0.05) identified between pHGG (n=64) and normal brain (n=20).
D. RT-PCR of indicated differential splicing events in 3 matched pairs of pHGG and normal brain.
E. Venn diagram comparing differentially expressed ($|\log2FC|>1$, adjusted $p<0.05$) and differentially spliced genes in pHGG.
F. Venn diagram comparing genes identified with differential ASE in pHGG by rMATS and SUPPA2.
G. Cytoscape network of significant Reactome pathways (FDR<0.05) enriched in differential ASE identified by SUPPA2.

Statistical tests: hypergeometric (F). Source data are provided as a Source Data file.

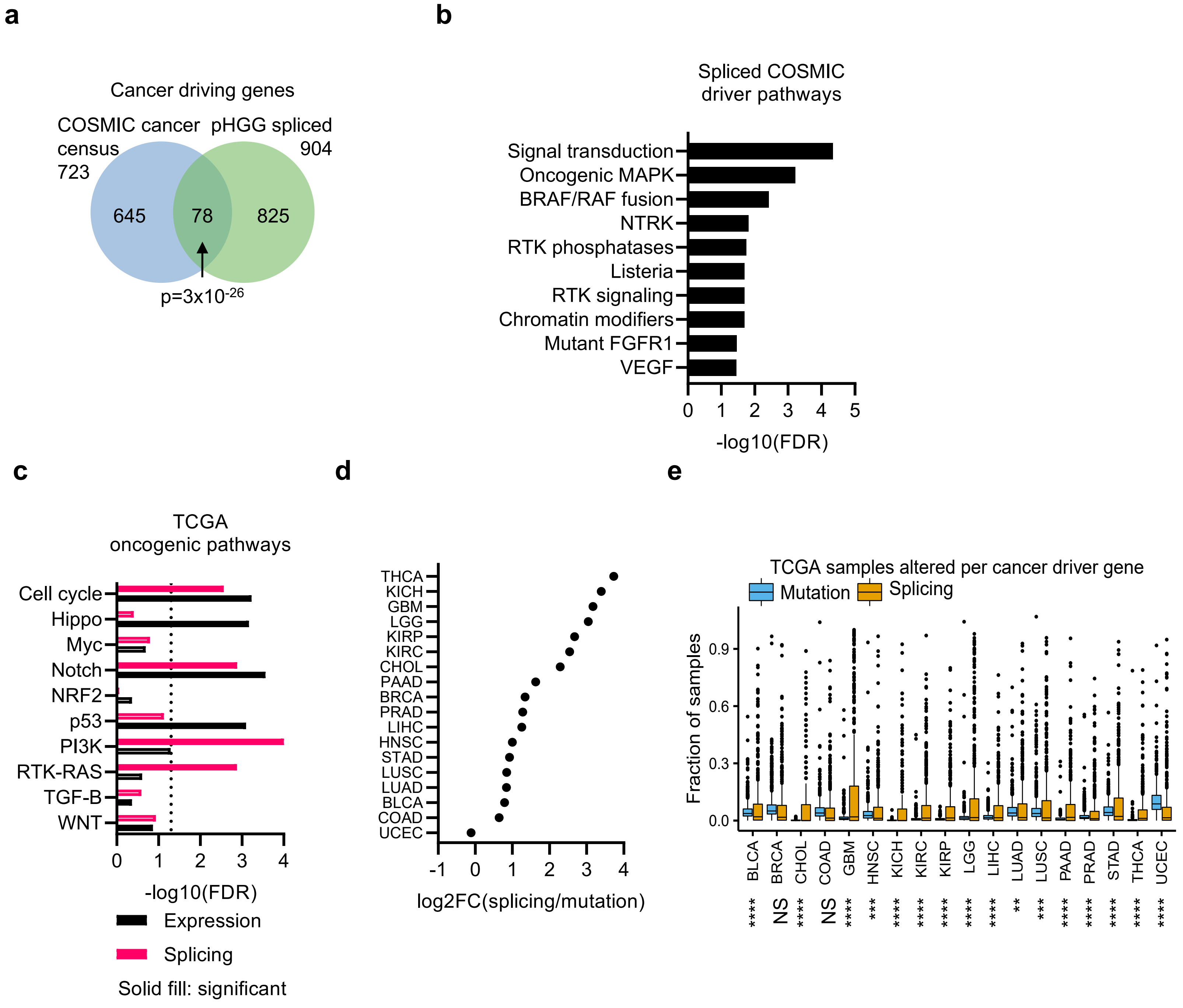

a
Cancer driving genes
COSMIC cancer census 723
pHGG spliced 904
645
78
825
p=3x10-26
b
Spliced COSMIC driver pathways
Signal transduction
Oncogenic MAPK
BRAF/RAF fusion
NTRK
RTK phosphatases
Listeria
RTK signaling
Chromatin modifiers
Mutant FGFR1
VEGF
0 1 2 3 4 5
-log10(FDR)
c
TCGA oncogenic pathways
Cell cycle
Hippo
Myc
Notch
NRF2
p53
PI3K
RTK-RAS
TGF-B
WNT
0 1 2 3 4
-log10(FDR)
Expression
Splicing
Solid fill: significant
d
THCA
KICH
GBM
LGG
KIRP
KIRC
CHOL
PAAD
BRCA
PRAD
LIHC
HNSC
STAD
LUSC
LUAD
BLCA
COAD
UCEC
-1 0 1 2 3 4
log2FC(splicing/mutation)
e
TCGA samples altered per cancer driver gene
Mutation
Splicing
Fraction of samples
0.0 0.3 0.6 0.9
BLCA ****
BRCA NS
CHOL ****
COAD NS
GBM ****
HNSC ***
KICH ****
KIRC ****
KIRP ****
LGG ****
LIHC ****
LUAD **
LUSC ***
PAAD ****
PRAD ****
STAD ****
THCA ****
UCEC ****

Supplementary Figure 3. pHGG and adult pan-cancer alternative splicing

A. Venn diagram comparing differentially spliced genes in pHGG with the COSMIC cancer driver gene census.
B. Reactome pathways enriched among differentially spliced COSMIC driver genes.
C. Gene set enrichment analysis (GSEA) was conducted on splicing and expression changes in TCGA oncogenic pathways by comparing pHGG (n=64) and normal brain (n=20). Solid-filled bars are significant with FDR<0.05. The dotted line marks the -log10(0.05) significance cutoff.
D. Plot of log2-fold change (log2FC) in mean splicing versus mutation burden across TCGA subtypes.
E. Pan-cancer burden of splicing and mutations in COSMIC cancer driver genes, showing the fraction of samples with an alteration per driver gene. The box marks the interquartile range (IQR) and shows the median value. The whiskers extend to 1.5x IQR and outliers outside this are plotted separately. See Supplementary Data 6 for n and p-values.

Statistical tests: hypergeometric (A), t-test with Benjamini-Hochberg multiple hypothesis testing correction (E). **: $p<0.01$, ***: $p<0.001$, ****: $p<0.0001$. NS: not significant. Source data are provided as a Source Data file.

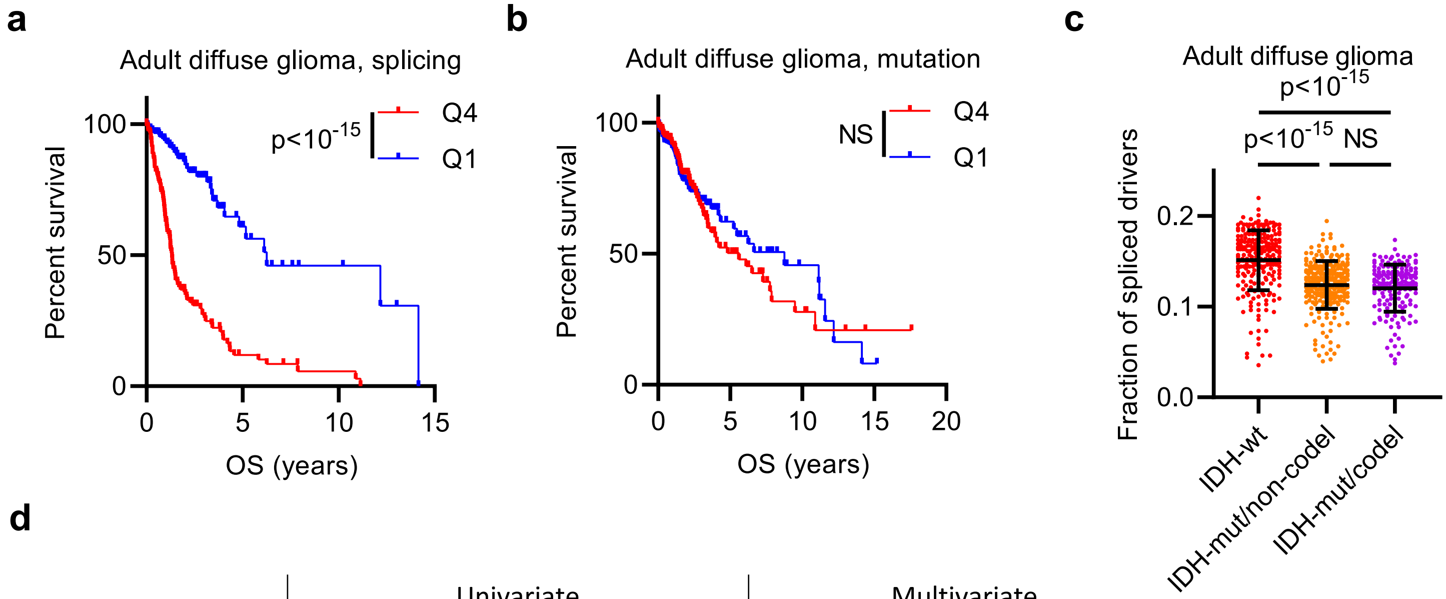

d

Adult diffuse glioma	Univariate			Multivariate		
	HR	95% CI	p-value	HR	95% CI	p-value
Splicing Q4 v Q1	4.93	3.32-7.32	$2.86x10^{-15}$	2.29	1.22-4.31	0.01
Mutation Q4 v Q1	1.11	0.77-1.61	0.58	1.01	0.57-1.80	0.98
IDH-mut/codel						
v IDH-mut/non-codel	1.55	0.95-2.53	0.082	1.80	0.57-5.64	0.31
v IDH-wt	13.28	8.43-20.92	$<2x10^{-16}$	10.08	3.40-29.88	$3.1x10^{-5}$

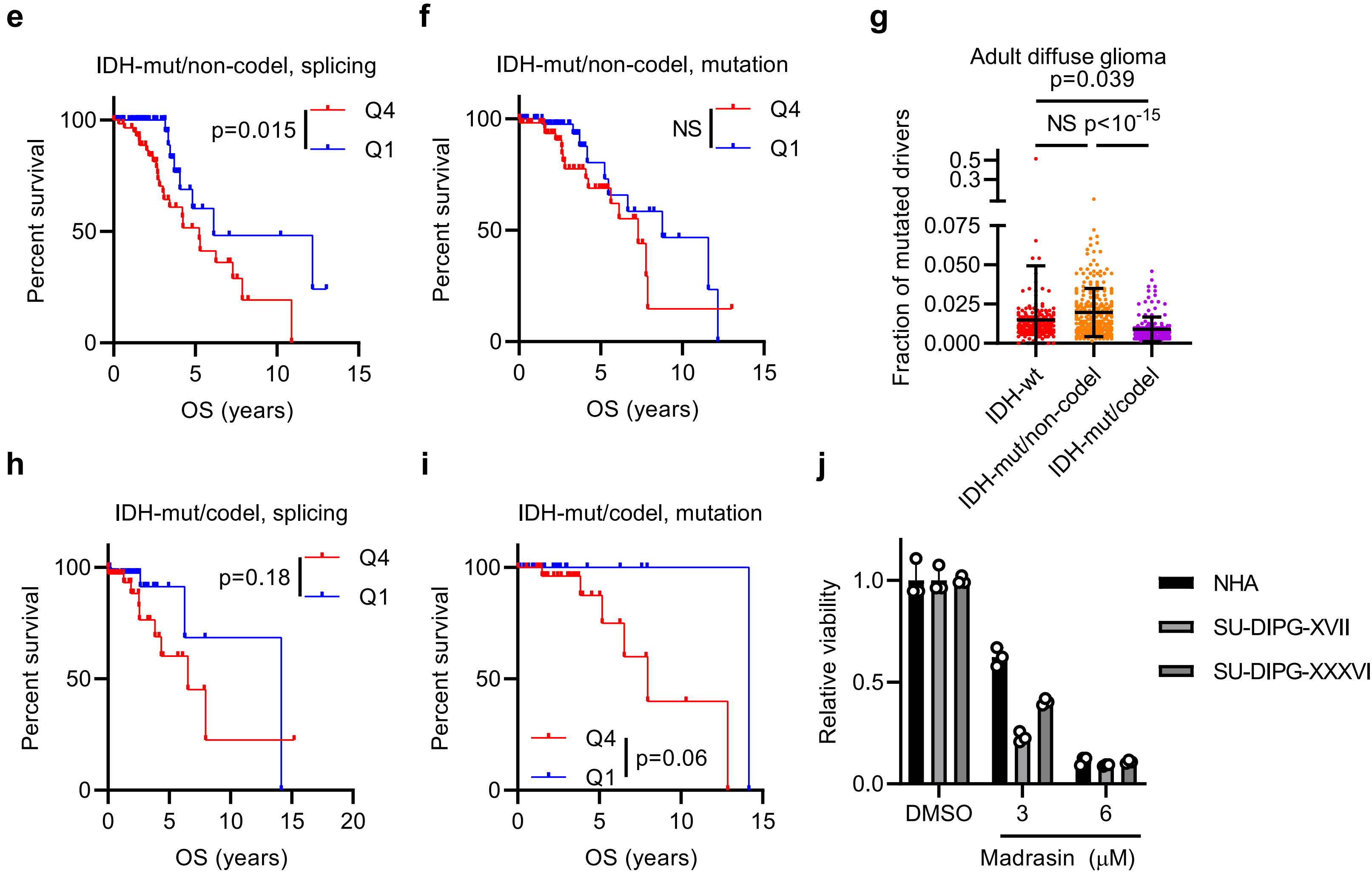

Supplementary Figure 4. Increased cancer driver splicing burden is associated with poor outcome in adult diffuse glioma.

A. Kaplan-Meier survival plot of adult diffuse glioma patients that are in the top (Q4, n=156) or bottom (Q1, n=160) quartile of splicing burden of cancer driver genes.
B. Kaplan-Meier survival plot of adult diffuse glioma patients that are in the top (Q4, n=158) or bottom (Q1, n=227) quartile of mutation burden of cancer driver genes.
C. Burden of cancer driver splicing alterations per sample in IDH-wt (n=231), IDH-mut/non-codel (n=257) and IDH-mut/codel (n=169) adult diffuse glioma. Bars show mean ± standard deviation.
D. Univariate and multivariate Cox proportional-hazards survival analysis of adult diffuse glioma accounting for cancer driver gene splicing and mutation burden (Q4 vs Q1), and subgroup. HR: hazard ratio. CI: confidence interval.
E. Kaplan-Meier survival plot of IDH-mut/non-codel patients that are in the top (Q4, n=60) or bottom (Q1, n=57) quartile of splicing burden of cancer driver genes.
F. Kaplan-Meier survival plot of IDH-mut/non-codel patients that are in the top (Q4, n=59) or bottom (Q1, n=59) quartile of mutation burden of cancer driver genes.
G. Burden of cancer driver mutations per sample in IDH-wt (n=225), IDH-mut/non-codel (n=253) and IDH-mut/codel (n=167) adult diffuse glioma. Bars show mean ± standard deviation.
H. Kaplan-Meier survival plot of IDH-mut/codel patients that are in the top (Q4, n=36) or bottom (Q1, n=42) quartile of splicing burden of cancer driver genes.
I. Kaplan-Meier survival plot of IDH-mut/codel patients that are in the top (Q4, n=33) or bottom (Q1, n=22) quartile of mutation burden of cancer driver genes.
J. Cell lines were treated with DMSO or madrasin for 4 days and viable cells counted (n=3). Bars show mean ± standard deviation.

Statistical tests: log-rank (A, B, E, F, H, I), ANOVA with Games-Howell's multiple comparisons test (C, G), Cox PH (D). Source data are provided as a Source Data file.

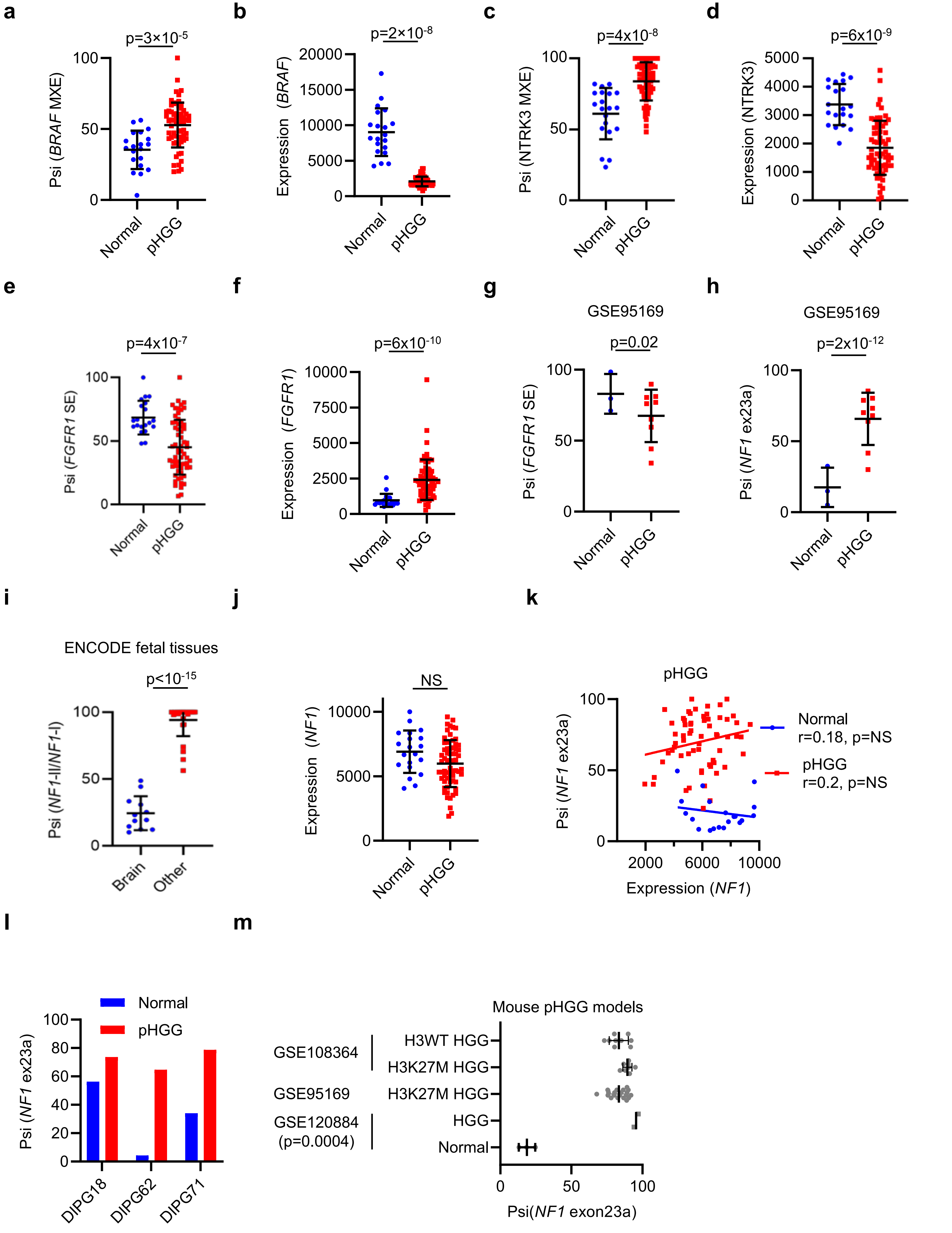

a
p=3×10-5
Psi (BRAF MXE)
Normal
pHGG
b
p=2×10-8
Expression (BRAF)
c
p=4x10-8
Psi (NTRK3 MXE)
d
p=6x10-9
Expression (NTRK3)
e
p=4x10-7
Psi (FGFR1 SE)
f
p=6x10-10
Expression (FGFR1)
g
GSE95169
p=0.02
Psi (FGFR1 SE)
h
GSE95169
p=2x10-12
Psi (NF1 ex23a)
i
ENCODE fetal tissues
p<10-15
Psi (NF1-II/NF1-I)
Brain
Other
j
NS
Expression (NF1)
k
pHGG
Normal r=0.18, p=NS
pHGG r=0.2, p=NS
Psi (NF1 ex23a)
Expression (NF1)
l
Normal
pHGG
Psi (NF1 ex23a)
DIPG18
DIPG62
DIPG71
m
Mouse pHGG models
GSE108364
H3WT HGG
H3K27M HGG
GSE95169
H3K27M HGG
GSE120884 (p=0.0004)
HGG
Normal
Psi(NF1 exon23a)

Supplementary Figure 5. AS of RAS/MAPK pathway genes in pHGG

A. Percent inclusion (Psi) of differential *BRAF* MXE (mutually exclusive exon) in pHGG (n=54) and normal brain (n=20). Bars show mean ± standard deviation.
B. Expression of *BRAF* in pHGG (n=64) and normal brain (n=20). Bars show mean ± standard deviation.
C. Psi of differential *NTRK3* MXE in pHGG (n=64) and normal brain (n=20). Bars show mean ± standard deviation.
D. Expression of *NTRK3* in pHGG (n=64) and normal brain (n=20). Bars show mean ± standard deviation.
E. Percent inclusion of differential *FGFR1* SE (skipped exon) in pHGG (n=64) and normal brain (n=20). Bars show mean ± standard deviation.
F. Expression of *FGFR1* in pHGG (n=64) and normal brain (n=20). Bars show mean ± standard deviation.
G. Psi of differential *FGFR1* SE in pHGG (n=9) and normal brain (n=3) from GSE95169[25]. Bars show mean ± standard deviation.
H. Psi of *NF1* exon23a in pHGG (n=9) and normal brain (n=3) from GSE95169[25]. Bars show mean ± standard deviation.
I. Percent inclusion (psi) of *NF1* exon23a in ENCODE RNA-Seq fetal tissue datasets from ENCODE. Samples were separated into brain (cerebellum, diencephalon, frontal cortex, occipital lobe, parietal lobe, temporal lobe; n=12) and non-brain (bladder, eye, heart, heart, kidney, liver, lung, skeletal muscle, skin, stomach, thyroid, tongue, umbilical cord, uterus; n=28). Bars show mean ± standard deviation.
J. *NF1* gene expression in pHGG (n=64) and normal brain (n=20). Bars show mean ± standard deviation.
K. Scatter plot of *NF1* expression and *NF1* exon23a inclusion in pHGG and normal brain. r: Pearson correlation.
L. qRT-PCR analysis of *NF1* exon23a inclusion in 3 matched pairs of pHGG and normal brain. Results are presented as the mean of 3 technical replicates.
M. *NF1* exon23a inclusion in mouse pHGG models. GSE120884[30] (H3K27M (n=2) and normal brain (n=3); H3K27M, GSE95169[23]; H3K27M and H3WT, GSE108364[31]).

Statistical tests: t-test (all). Source data are provided as a Source Data file.

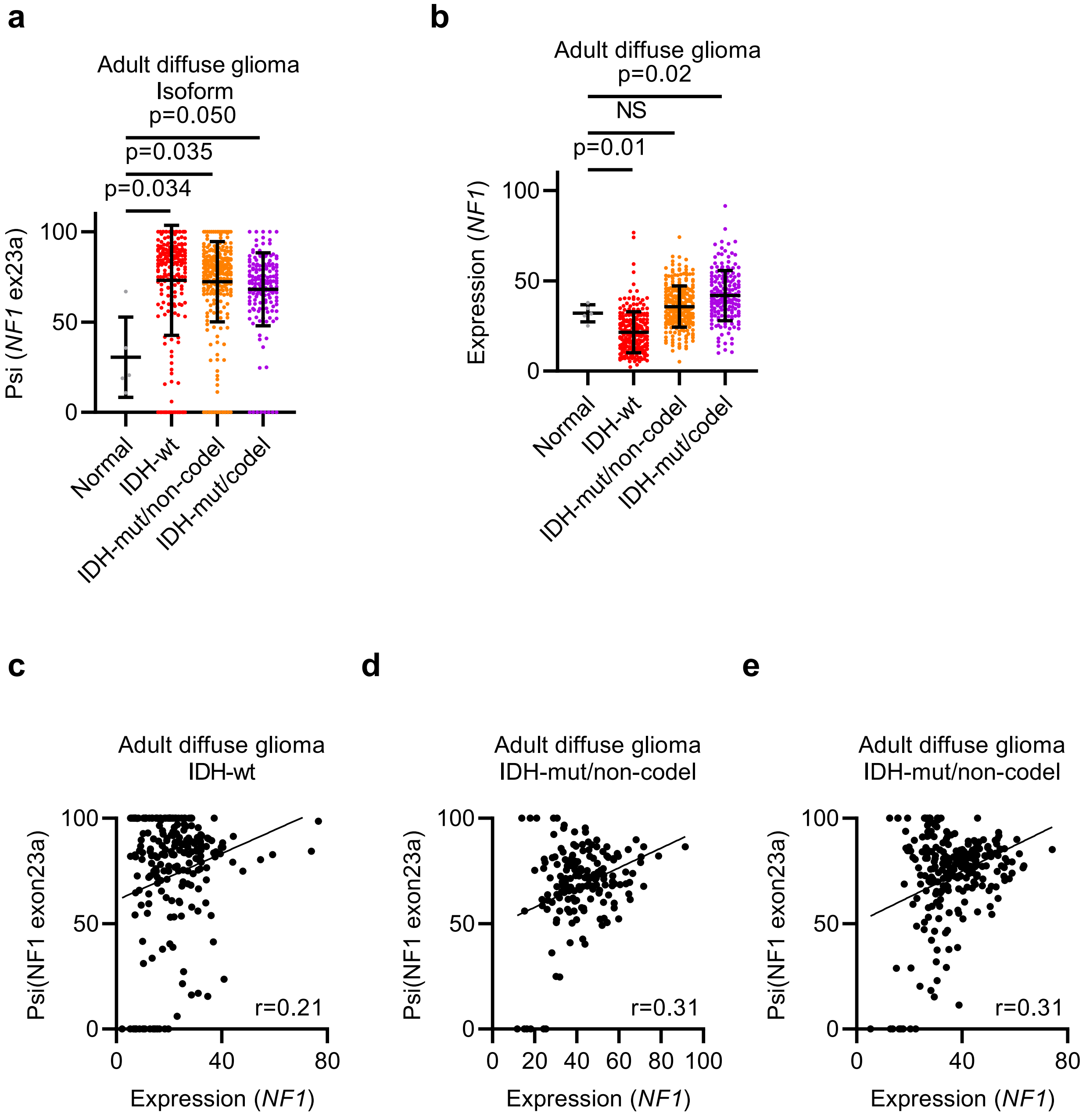

a
Adult diffuse glioma
Isoform
p=0.050
p=0.035
p=0.034
Psi (NF1 ex23a)
100
50
0
Normal
IDH-wt
IDH-mut/non-codel
IDH-mut/codel
b
Adult diffuse glioma
p=0.02
NS
p=0.01
Expression (NF1)
100
50
0
Normal
IDH-wt
IDH-mut/non-codel
IDH-mut/codel
c
Adult diffuse glioma
IDH-wt
Psi(NF1 exon23a)
r=0.21
Expression (NF1)
d
Adult diffuse glioma
IDH-mut/non-codel
Psi(NF1 exon23a)
r=0.31
Expression (NF1)
e
Adult diffuse glioma
IDH-mut/non-codel
Psi(NF1 exon23a)
r=0.31
Expression (NF1)

Supplementary Figure 6. Alternative splicing of *NF1* in adult HGG

A. *NF1* exon23a percent inclusion (psi) based on NF1 transcript ratios in TCGA diffuse glioma dataset: adult normal brain (n=5) and IDH-wt (n=231), IDH-mut/non-codel (n=256) and IDH-mut/codel (n=170) HGG. Bars show mean ± standard deviation.
B. *NF1* expression in normal brain (n=5) and IDH-wt (n=233), IDH-mut/non-codel (n=258) and IDH-mut/codel (n=172) adult diffuse glioma subgroups. Bars show mean ± standard deviation.
C. Scatter plot of *NF1* expression and percent inclusion (psi) of *NF1* exon23a in IDT-wt adult diffuse glioma. r: Pearson correlation.
D. Scatter plot of *NF1* expression and percent inclusion (psi) of *NF1* exon23a in IDT-mut/non-codel adult diffuse glioma. r: Pearson correlation.
E. Scatter plot of *NF1* expression and percent inclusion (psi) of *NF1* exon23a in IDT-mut/codel adult diffuse glioma. r: Pearson correlation.

Statistical tests: Brown-Forsythe ANOVA with Dunnett’s T3 multiple comparisons test (A, B).
Source data are provided as a Source Data file.

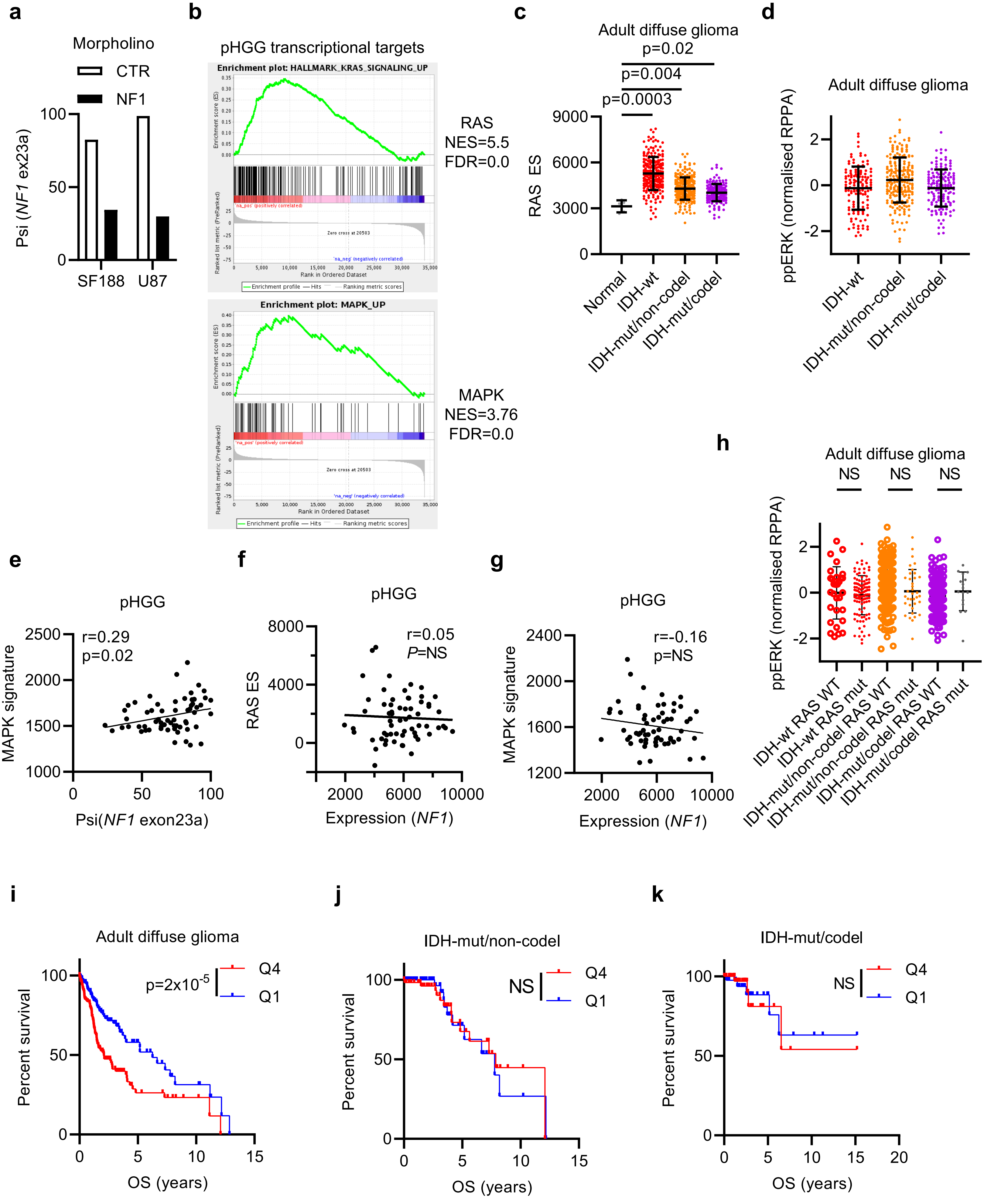
a
Morpholino
CTR
NF1
Psi (NF1 ex23a)
100
50
0
SF188
U87
b
pHGG transcriptional targets
Enrichment plot: HALLMARK_KRAS_SIGNALING_UP
RAS
NES=5.5
FDR=0.0
Enrichment plot: MAPK_UP
MAPK
NES=3.76
FDR=0.0
Enrichment score (ES)
Ranked list metric (PreRanked)
Rank in Ordered Dataset
Zero cross at 20503
'na_pos' (positively correlated)
'na_neg' (negatively correlated)
Enrichment profile — Hits — Ranking metric scores
c
Adult diffuse glioma
p=0.02
p=0.004
p=0.0003
RAS ES
9000
6000
3000
0
Normal
IDH-wt
IDH-mut/non-codel
IDH-mut/codel
d
Adult diffuse glioma
ppERK (normalised RPPA)
2
0
-2
IDH-wt
IDH-mut/non-codel
IDH-mut/codel
e
pHGG
r=0.29
p=0.02
MAPK signature
2500
2000
1500
1000
0
50
100
Psi(NF1 exon23a)
f
pHGG
r=0.05
P=NS
RAS ES
8000
4000
0
2000
6000
10000
Expression (NF1)
g
pHGG
r=-0.16
p=NS
MAPK signature
2400
2000
1600
1200
2000
6000
10000
Expression (NF1)
h
Adult diffuse glioma
NS
NS
NS
ppERK (normalised RPPA)
2
0
-2
IDH-wt RAS WT
IDH-wt RAS mut
IDH-mut/non-codel RAS WT
IDH-mut/non-codel RAS mut
IDH-mut/codel RAS WT
IDH-mut/codel RAS mut
i
Adult diffuse glioma
p=2x10-5
Q4
Q1
Percent survival
100
50
0
0
5
10
15
OS (years)
j
IDH-mut/non-codel
NS
Q4
Q1
Percent survival
100
50
0
0
5
10
15
OS (years)
k
IDH-mut/codel
NS
Q4
Q1
Percent survival
100
50
0
0
5
10
15
20
OS (years)

Supplementary Figure 7. Regulation of RAS/MAPK by *NF1* exon23a in HGG

A. qRT-PCR analysis of *NF1* exon23a inclusion in cells harvested 40 hours post-transfection with control or morpholinos targeting the up and downstream intron/exon junctions of *NF1* exon23a. Results are representative of two experiments.
B. Gene set enrichment analysis of RAS signature genes and MAPK signature genes[68] in pHGG vs normal brain. FDR: false-discovery rate. NES: normalised enrichment score.
C. RAS pathway activation single sample gene set enrichment analysis (ssGSEA) of normal brain (n=5) and IDH-wt (n=243), IDH-mut/non-codel (n=270) and IDH-mut/codel (n=173) adult diffuse glioma. Bars show mean ± standard deviation. ES: enrichment score.
D. Normalised reverse-phase protein array (RPPA) signal for phospho-ERK (ppERK) for IDH-wt (n=100), IDH-mut/non-codel (n=141) and IDH-mut/codel (n=74) adult diffuse glioma. Bars show mean ± standard deviation.
E. Scatter plot of *NF1* exon23a psi and MAPK signature score in pHGG. r: Pearson correlation.
F. Scatter plot of *NF1* expression and RAS ES in pHGG (enrichment score, determined from ssGSEA). r: Pearson correlation.
G. Scatter plot of *NF1* expression and MAPK signature score in pHGG. r: Pearson correlation.
H. Normalised RPPA ppERK signal for adult diffuse glioma that are RAS/MAPK WT or mutant. n: IDH-wt WT=30, mut=102; IDH-mut/non-codel WT=170, mut=36; IDH-mut/codel WT=130, mut=16. Bars show mean ± standard deviation.
I. Kaplan-Meier survival plot comparing Q4 and Q1 patients for all adult diffuse glioma (n: Q1=166, Q4=159).
J. Kaplan-Meier survival plot comparing Q4 and Q1 patients for IDH-mut/non-codel adult diffuse glioma (n: Q1=68, Q4=66).
K. Kaplan-Meier survival plot comparing Q4 and Q1 patients for IDH-mut/codel adult diffuse glioma (n: Q1=42, Q4=43).

Statistical tests: Brown-Forsythe ANOVA with Dunnett's T3 multiple comparisons test (C), t (E, F, G, H), Gehan-Breslow-Wilcoxon (I, J, K). NS: not significant. Source data are provided as a Source Data file.

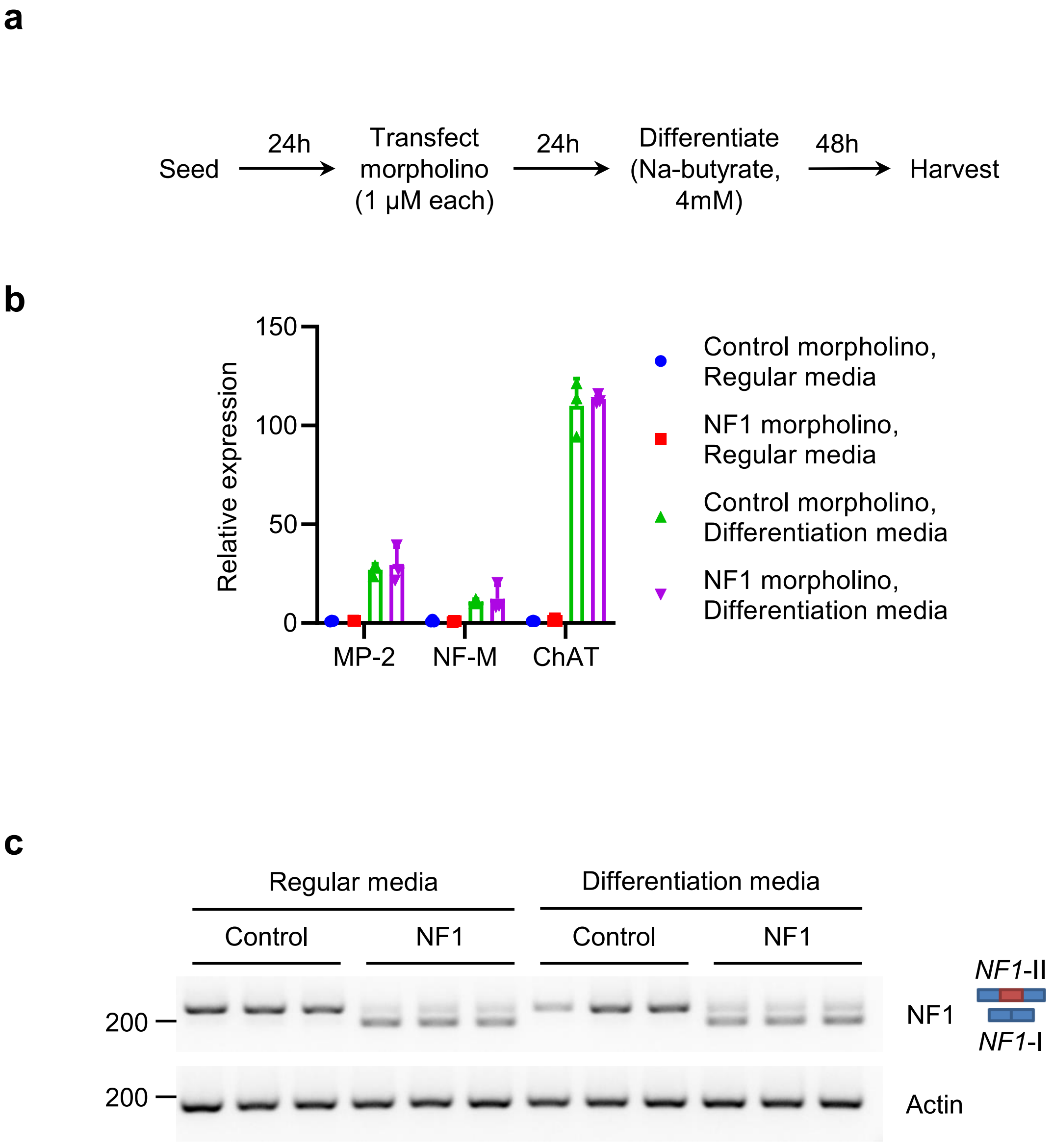
a
Seed
24h
Transfect morpholino (1 μM each)
24h
Differentiate (Na-butyrate, 4mM)
48h
Harvest
b
Relative expression
150
100
50
0
MP-2
NF-M
ChAT
Control morpholino, Regular media
NF1 morpholino, Regular media
Control morpholino, Differentiation media
NF1 morpholino, Differentiation media
c
Regular media
Differentiation media
Control
NF1
Control
NF1
200
200
NF1
Actin
NF1-II
NF1-I

Supplementary Figure 8. *NF1* exon23a splicing in HGG differentiation

A. Schematic of morpholino transfection and differentiation method.
B. U87 cells were transfected with control or NF1-specific morpholinos and differentiated according to A (n=3 independent experiments). Expression of neuronal marker genes was measured by quantitative real-time PCR (qRT-PCR), normalised to *ACTB* levels. Bars show mean ± standard deviation.
C. RT-PCR of *NF1* exon23a inclusion in cells from B. Statistical tests: t (B). Source data are provided as a Source Data file.

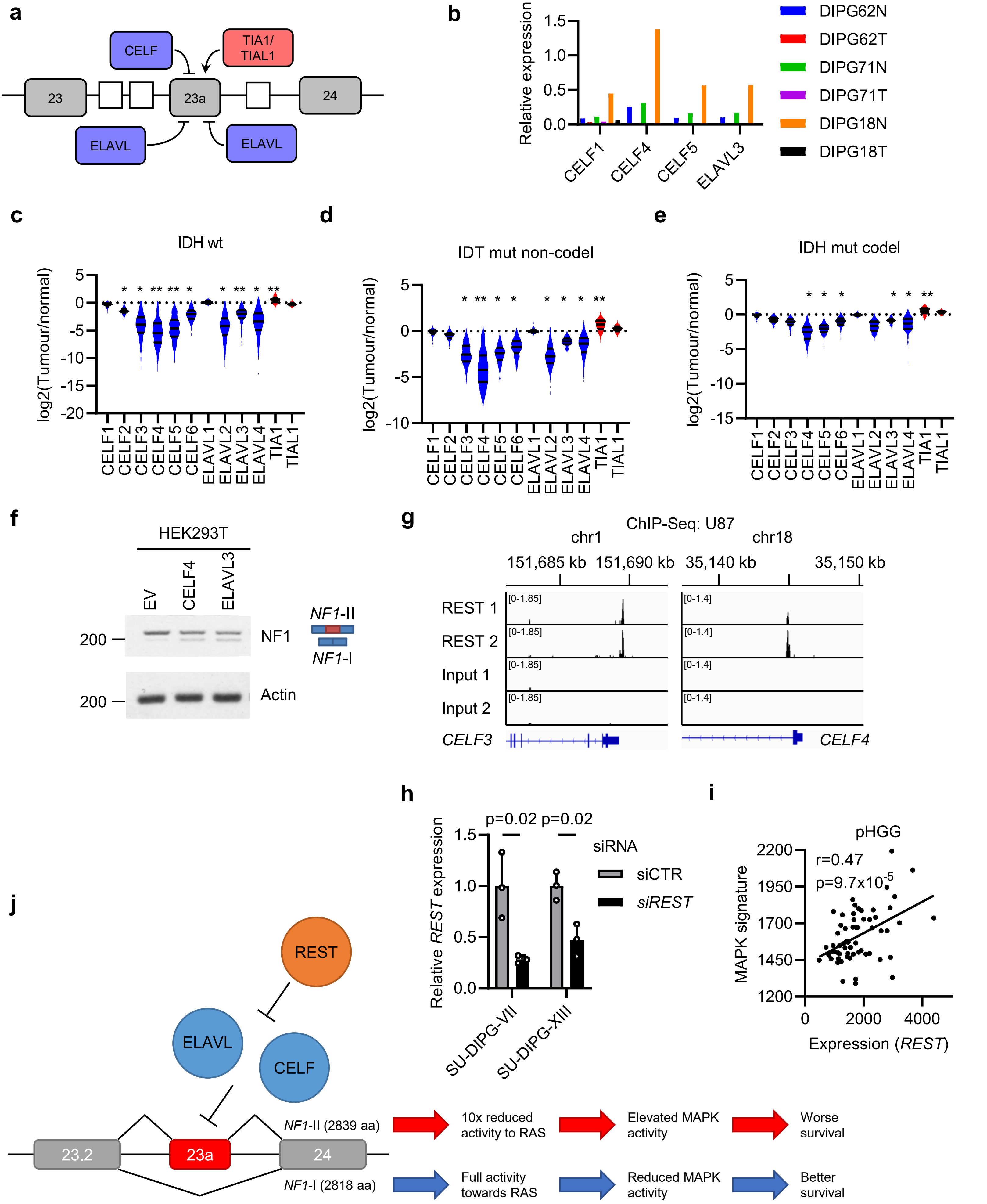

a
CELF
TIA1/ TIAL1
23
23a
24
ELAVL
ELAVL
b
Relative expression
1.5
1.0
0.5
0.0
CELF1
CELF4
CELF5
ELAVL3
DIPG62N
DIPG62T
DIPG71N
DIPG71T
DIPG18N
DIPG18T
c
IDH wt
log2(Tumour/normal)
5
0
-5
-10
-15
-20
CELF1
CELF2
CELF3
CELF4
CELF5
CELF6
ELAVL1
ELAVL2
ELAVL3
ELAVL4
TIA1
TIAL1
d
IDT mut non-codel
log2(Tumour/normal)
e
IDH mut codel
log2(Tumour/normal)
f
HEK293T
EV
CELF4
ELAVL3
200 —
NF1
200 —
Actin
NF1-II
NF1-I
g
ChIP-Seq: U87
chr1
chr18
151,685 kb
151,690 kb
35,140 kb
35,150 kb
REST 1
REST 2
Input 1
Input 2
[0-1.85]
[0-1.4]
CELF3
CELF4
h
p=0.02
p=0.02
Relative REST expression
siRNA
siCTR
siREST
SU-DIPG-VII
SU-DIPG-XIII
i
pHGG
r=0.47
p=9.7x10-5
MAPK signature
2200
1950
1700
1450
1200
0
2000
4000
Expression (REST)
j
REST
ELAVL
CELF
23.2
23a
24
NF1-II (2839 aa)
NF1-I (2818 aa)
10x reduced activity to RAS
Elevated MAPK activity
Worse survival
Full activity towards RAS
Reduced MAPK activity
Better survival

Supplementary Figure 9. Regulation of *NF1* exon23a splicing in HGG

A. Schematic of *NF1* exon23a regulation by negative (blue: CELF, ELAVL) and positive (red: TIA1/TIAL1) families of splice factors, showing relative binding sites of each family (white box).
B. qRT-PCR analysis of gene expression in matched pHGG/normal brain, relative to *ACTB* in 3 matched pairs of pHGG and normal brain. Results are presented as the mean of 3 technical replicates.
C. Expression ratio (log2 fold change) of negative (blue) and positive (red) *NF1* exon23a splicing regulators between IDH-wt adult diffuse glioma and normal brain. See Supplementary Data 6 for p-values.
D. Expression ratio (log2 fold change) of negative (blue) and positive (red) *NF1* exon23a splicing regulators between IDH-mut/non-codel adult diffuse glioma and normal brain. See Supplementary Data 6 for p-values.
E. Expression ratio (log2 fold change) of negative (blue) and positive (red) *NF1* exon23a splicing regulators between IDH-mut/codel adult diffuse glioma and normal brain. See Supplementary Data 6 for p-values.
F. HEK293T cells transduced with control (EV) lentivirus or expressing CELF4 or ELAVL3 were analysed by RT-PCR. Bands corresponding to *NF1*-II (includes exon23a, red box) and *NF1*-I are shown at the left. Gels are representative of 2 independent experiments.
G. Genome browser screenshots from two replicates of REST ChIP-Seq and input control from U87 cells (ENCODE).
H. qRT-PCR in SU-DIPG-VII and SU-DIPG-XIII cells transfected with control or *REST*-specific siRNA. Bars show mean ± standard deviation (n=3 experiments) relative to 18S housekeeping, normalised to siCTR expression for each gene.
I. Scatter plot of *REST* expression and MAPK activity score in pHGG. r: Pearson correlation.
J. Model depicting NF1 exon23a-mediated modulation of RAS activity in high-grade glioma.

Statistical tests: t-test (all). *: $p<0.05$, **: $p<0.01$. Source data are provided as a Source Data file.

Supplementary Tables

Supplementary Table 1: Morpholino sequences used in the study.

Morpholino	Sequence (5'-3')
intron30/exon31(exon23a)	TGCCTACAGAACAGAGATGAGCACA
exon31(exon23a)/intron31	GTTCTCCAAACTTACTGATTTTTTG
Non-targeting control	CCTCTTACCTCAGTTACAATTTATA

Supplementary Table 2: Primer sequences used in the study.

Oligo	Sequence (5'-3')	Use
pCDH_CELF4 forward	ACCTCCATAGAAGATTCTAGAGCCAC CATGTATATAAAGATGGCCACGTTA	Cloning
pCDH_CELF4 reverse	GTCGTCCTTGTAGTCGGATCCGTACG GGCGATTGGCGTCTTT	Cloning
pCDH_ELAVL3 forward	ACCTCCATAGAAGATTCTAGAGCCAC CATGGTCACTCAGATACTGGGGG	Cloning
pCDH_ELAVL3 reverse	GTCGTCCTTGTAGTCGGATCCCGCCTT GTGCTGTTTGCTGGT	Cloning
NF1_ex23.1 forward	TGATCACATCCTCTGATTGGCA	RT-PCR
NF1_ex23.2 forward	CCATGCCATCATCAGTTCCT	qRT-PCR
NF1_ex23a reverse	ATTCAGTAGGGAGTGGCAAGTT	qRT-PCR
NF1_ex24 reverse	GAGGGAAACGCTGGCTAAC	RT-PCR
NF1_ex23.2-ex24 reverse	GCTGGCTAACCACCTGGTATAAA	qRT-PCR
ATP2B1 forward	TCGGCATCAGTGTCATCAAT	RT-PCR
ATP2B1 reverse	GTGGCCAAATCTTGTGGTTT	RT-PCR
MADD_26 forward	AGATGTGAGCCAGCGAGTCT	RT-PCR
MADD_26 reverse	GGTCCATACCCATCCCTTCT	RT-PCR
MDB1 forward	AGCCATCTTGGAGTCACAGC	RT-PCR
MBD1 reverse	CAGACCCAAGAGGATTGTGG	RT-PCR
SMARCC2 forward	GCCCAAAGTTTTGCATGTCT	RT-PCR
SMARCC2 reverse	CATGTCTTGGCTGACACACC	RT-PCR
FGFR1 forward	CATACGGTTTGGTTTGGTGTT	RT-PCR
FGFR1 reverse	CACACTCTGCACCGCTAGG	RT-PCR
TPD52 forward	TTTTCGACCTTTTCTTCAAATG	RT-PCR
TPD52 reverse	TATCCCAGGCTGGACAGAAG	RT-PCR
ERC1 forward	CTTCAAATCCTCCCATTCCA	RT-PCR
ERC1 reverse	GGAAGCTGGTGGAGTGAGTC	RT-PCR
ABI2 forward	CTCCTCCCAGTGTCTTTCCA	RT-PCR
ABI2 reverse	GCAGGCCTATTCATGCTGTA	RT-PCR
MP-2 forward	AGCAGTCCTGAAAGGTGAACA	qRT-PCR
MP-2 reverse	ATCGTGGAACTCCATCTTCG	qRT-PCR
NF-M forward	TCAACGTCAAGATGGCTCTG	qRT-PCR
NF-M reverse	CTTCCACCTTGGGTTTCTGA	qRT-PCR

ChAT forward	CTGCCCCACCAGTACGTC	qRT-PCR
ChAT reverse	CATGGACACGGGTTACAGTG	qRT-PCR
CELF1 forward	ACCCCATAGCCTCACTTGG	qRT-PCR
CELF1 reverse	CCATTTAAAGCAGCCATTCC	qRT-PCR
CELF3 forward	AGCTAAAGCGGCCTAAGGAT	qRT-PCR
CELF3 reverse	CGTGGGAAGAGGGTGTGA	qRT-PCR
CELF4 forward	CCAATCGCCCGTACTGAG	qRT-PCR
CELF4 reverse	TTAATGTAGCCCGTTCAGCAT	qRT-PCR
CELF5 forward	CTTGCATGGGAGCCAGAC	qRT-PCR
CELF5 reverse	GTCGGCGAACTTGACCAC	qRT-PCR
ELAVL3 forward	ACGGCCTCAAATTACAGACG	qRT-PCR
ELAVL3 reverse	CGCTGACGTACAGGTTAGCAT	qRT-PCR
ACTB forward	TCCCTGGAGAAGAGCTACGA	RT-PCR/qRT-PCR
ACTB reverse	AGCACTGTGTTGGCGTACAG	RT-PCR/qRT-PCR
18S forward	CGGCGACGACCCATTCGAAC	qRT-PCR
18S reverse	GAATCGAACCCTGATTCCCCGTC	qRT-PCR
CELF3 forward	GAGCCACGCCCTCTTTCTAT	ChIP
CELF3 reverse	ACCCCACTTTAGGGTTCCTCT	ChIP
CELF4 forward	GTATTTTCAGCACCACGGCG	ChIP
CELF4 reverse	ACAGCGACCTGAAAGATGGG	ChIP
ELAVL3 forward	CATGCTCGCTCTCTGCTAGG	ChIP
ELAVL3 reverse	CCAGTGTGTGCTAGTTCCCG	ChIP
