# Supplementary material for: Splicing is an alternate oncogenic pathway activation mechanism in glioma
Source: Nat Commun. 2022 Jan 31;13:588. doi: 10.1038/s41467-022-28253-4 (PMC8803922; doi:10.1038/s41467-022-28253-4)
Supplement: Supplementary file 2 — Description of Additional Supplementary Files [file 41467_2022_28253_MOESM2_ESM.pdf]

Description of Additional Supplementary Files

File Name: Supplementary Data 1
Description: Patient cohort

File Name: Supplementary Data 2
Description: Core RAS/MAPK genes, spliceosome genes, spliceosome alterations

File Name: Supplementary Data 3
Description: Differential ASE in pHGG vs normal brain

File Name: Supplementary Data 4
Description: sample numbers and p-values for Figure 2f, Supplementary Figures 3e, 9c-e
