# Supplementary material for: Splicing is an alternate oncogenic pathway activation mechanism in glioma
Source: Nat Commun. 2022 Jan 31;13:588. doi: 10.1038/s41467-022-28253-4 (PMC8803922; doi:10.1038/s41467-022-28253-4)
Supplement: Supplementary file 7 — Reporting Summary [file 41467_2022_28253_MOESM7_ESM.pdf]

nature research

Corresponding author(s): Cynthia Hawkins

Last updated by author(s): Jan 7, 2022

Reporting Summary

Nature Research wishes to improve the reproducibility of the work that we publish. This form provides structure for consistency and transparency in reporting. For further information on Nature Research policies, see our Editorial Policies and the Editorial Policy Checklist.

Statistics

For all statistical analyses, confirm that the following items are present in the figure legend, table legend, main text, or Methods section.

n/a	Confirmed	
☐	☒	The exact sample size (*n*) for each experimental group/condition, given as a discrete number and unit of measurement
☐	☒	A statement on whether measurements were taken from distinct samples or whether the same sample was measured repeatedly
☐	☒	The statistical test(s) used AND whether they are one- or two-sided<br>*Only common tests should be described solely by name; describe more complex techniques in the Methods section.*
☐	☒	A description of all covariates tested
☐	☒	A description of any assumptions or corrections, such as tests of normality and adjustment for multiple comparisons
☐	☒	A full description of the statistical parameters including central tendency (e.g. means) or other basic estimates (e.g. regression coefficient) AND variation (e.g. standard deviation) or associated estimates of uncertainty (e.g. confidence intervals)
☐	☒	For null hypothesis testing, the test statistic (e.g. *F*, *t*, *r*) with confidence intervals, effect sizes, degrees of freedom and *P* value noted<br>*Give P values as exact values whenever suitable.*
☒	☐	For Bayesian analysis, information on the choice of priors and Markov chain Monte Carlo settings
☒	☐	For hierarchical and complex designs, identification of the appropriate level for tests and full reporting of outcomes
☐	☒	Estimates of effect sizes (e.g. Cohen's *d*, Pearson's *r*), indicating how they were calculated

Our web collection on statistics for biologists contains articles on many of the points above.

Software and code

Policy information about availability of computer code

Data collection	No software was used.
Data analysis	Trimmomatic-v0.32, bwa-v0.7.8, Picard-v2.5.0, GATK-v3.6.0, GATK-v4.0.1.2, VarScan-v2.3.6, SnpEFF-v4.3k, CNVkit v0.8.6, STAR v2.5.0, HTSeq, edgeR, DESeq, Rtsne-v0.15, GSEA-v3.0, ssSSEA (GenePattern), RSEM-v1.2, rMATS-v4.0.1, SUPPA2, rmats2sashimiplot (https://github.com/Xinglab/rmats2sashimiplot), Cytoscape-v3.8.2, ClueGO-v2.5.7, R-v3.6.1, GraphPad Prism 8, MEME Suite-v5.4.1

For manuscripts utilizing custom algorithms or software that are central to the research but not yet described in published literature, software must be made available to editors and reviewers. We strongly encourage code deposition in a community repository (e.g. GitHub). See the Nature Research guidelines for submitting code & software for further information.

Data

Policy information about availability of data

All manuscripts must include a data availability statement. This statement should provide the following information, where applicable:

- Accession codes, unique identifiers, or web links for publicly available datasets
- A list of figures that have associated raw data
- A description of any restrictions on data availability

The publicly available total RNA-Seq data from 9 pHGG and 3 normal brains are available from Gene Expression Omnibus (GEO) under accession number GSE95169 (https://www.ncbi.nlm.nih.gov/geo/query/acc.cgi?acc=GSE95169)23.
The publicly available mouse pHGG model RNA-Seq data are available from GEO under accession numbers GSE120884 https://www.ncbi.nlm.nih.gov/geo/query/acc.cgi?acc=GSE120884)30, GSE95169 (https://www.ncbi.nlm.nih.gov/geo/query/acc.cgi?acc=GSE95169)23 and GSE108364 (https://www.ncbi.nlm.nih.gov/geo/query/acc.cgi?acc=GSE108364)31.
The publicly available REST ChIP-Seq data are available from GEO under accession number GSE32465 (https://www.ncbi.nlm.nih.gov/geo/query/acc.cgi?

acc=GSE32465)43. TF binding sites in CELF/ELAVL gene promoters were identified with Enrichr (https://maayanlab.cloud/Enrichr/)75. RE1 motif-containing, REST-bound genes were identified from REST ChIP-Seq analysis44.
Level 3 processed adult diffuse glioma RNA-Seq (RSEM quantifications of genes and isoforms), RPPA and clinical data were downloaded from The Cancer Genome Atlas/Broad Firehose (https://gdac.broadinstitute.org/), and mutations and copy number variations in the same samples were assessed with cBioPortal (https://www.cbioportal.org/). Only primary tumours were considered4,7,59. TCGA RNA-Seq splicing data was retrieved from TCGA SpliceSeq (https://bioinformatics.mdanderson.org/TCGASpliceSeq/singlegene.jsp)74. CBTTC11,12 data was retrieved from PedcBioPortal (https://pedcbioportal.org/).
DNA methylation data are available in GEO under accession number GSE49822 (https://www.ncbi.nlm.nih.gov/geo/query/acc.cgi?acc=GSE49822) and GSE55712 (https://www.ncbi.nlm.nih.gov/geo/query/acc.cgi?acc=GSE55712)21,76. Probes associated with genes contained in either the KEGG spliceosome or Reactome mRNA splicing pathway ontologies (Fig. 1b) were analysed.
Previously published data on our cohort2,30 is deposited in GEO or the European Genomics Archive (EGA); SNP6.0 data are available in GEO under accession number GSE50024 (https://www.ncbi.nlm.nih.gov/geo/query/acc.cgi?acc=GSE50024); WGS data are available in EGA under accession number EGAS00001000575 (https://ega-archive.org/studies/EGAS00001000575); WES data are available in EGA under accession numbers EGAS00001000575 (https://ega-archive.org/studies/EGAS00001000575) and EGAD00001006450 (https://ega-archive.org/datasets/EGAD00001006450); RNA-Seq data are available in EGA under accession number EGAD00001006450 (https://ega-archive.org/datasets/EGAD00001006450).
The data newly generated on our cohort is deposited with EGA; WES data are available under accession number (https://ega-archive.org/datasets/EGAD00001008278); RNA-Seq data are available under accession EGAD00001008279 (https://ega-archive.org/datasets/EGAD00001008279). The data is available under controlled access to comply with data protection regulations, and can be accessed by application to the data access committee via CH (cynthia.hawkins@sickkids.ca). The remaining data are available within the Article, Supplementary Information or Source Data file.

Field-specific reporting

Please select the one below that is the best fit for your research. If you are not sure, read the appropriate sections before making your selection.

[x] Life sciences [ ] Behavioural & social sciences [ ] Ecological, evolutionary & environmental sciences

For a reference copy of the document with all sections, see nature.com/documents/nr-reporting-summary-flat.pdf

Life sciences study design

All studies must disclose on these points even when the disclosure is negative.

Sample size	Sample size was not pre-determined. NGS directly generated in the study was derived from patients presenting at the Hospital for Sick Children and after informed consent was obtained. Functional experiments were conducted with a minimum of 3 biological replicates unless otherwise stated.
Data exclusions	No data were excluded from analyses.
Replication	In vitro experiments were performed in triplicate unless otherwise stated. Replication information is included in the Figure Legends and/or text. All replication attempts were successful.
Randomization	Randomization was not applied because we studied differences between tissues of known origin ie tumour and normal
Blinding	We did not blind as the primary objective was to determine alternative splicing differences in tumours compared with normal brain, which required knowledge of the tissue type.

Reporting for specific materials, systems and methods

We require information from authors about some types of materials, experimental systems and methods used in many studies. Here, indicate whether each material, system or method listed is relevant to your study. If you are not sure if a list item applies to your research, read the appropriate section before selecting a response.

Materials & experimental systems

n/a	Involved in the study	
[ ]	[x]	Antibodies
[ ]	[x]	Eukaryotic cell lines
[x]	[ ]	Palaeontology and archaeology
[x]	[ ]	Animals and other organisms
[ ]	[x]	Human research participants
[x]	[ ]	Clinical data
[x]	[ ]	Dual use research of concern

Methods

n/a	Involved in the study	
[x]	[ ]	ChIP-seq
[x]	[ ]	Flow cytometry
[x]	[ ]	MRI-based neuroimaging

Antibodies

Antibodies used	ERK1/2 (Cell Signaling 9102)

	phospho-ERK1/2 (Cell Signaling Thr202/204; 9101)<br>COX-IV (clone 3E11; Cell Signaling 4850)<br>REST (Millipore, 07-579)<br>IgG (Santa Cruz, sc2027)
Validation	ERK1/2 (https://www.cellsignal.com/products/primary-antibodies/p44-42-mapk-erk1-2-antibody/9102)<br>phospho-ERK1/2 (https://www.cellsignal.com/products/primary-antibodies/phospho-p44-42-mapk-erk1-2-thr202-tyr204-antibody/9101)<br>COX-IV (https://www.cellsignal.com/products/primary-antibodies/cox-iv-3e11-rabbit-mab/4850)

Eukaryotic cell lines

Policy information about cell lines

Cell line source(s)	HEK293T, U87, U343: ATCC<br>Fetal-NHA: ABM<br>SF188: Chris Jones (Institute for Cancer Research, London; non-commerical original source)<br>SU-DIPG-IV, SU-DIPG-VI, SU-DIPG-VII, SU-DIPG-XIII, SU-DIPG-XVII and SU-DIPG-XXXVI : Michele Monje (Stanford University, California; non-commerical original source)
Authentication	Cell lines obtained from collaborators were authenticated by STR profiling. Commercial cells were not authenticated.
Mycoplasma contamination	All cell lines were routinely tested for mycoplasma. No contaminations were detected.
Commonly misidentified lines<br>(See ICLAC register)	HEKT293T cells were used for lentiviral production and simple transfection assays.

Human research participants

Policy information about studies involving human research participants

Population characteristics	Samples were taken from fully-consented patients diagnosed with diffuse midline or hemispheric glioma presenting to The Hospital for Sick Children. Their characteristics were: mean age 7.7 years, median age 6.9 years, 54% female.
Recruitment	Written consent from a legally authorised representative (all patients in this study were under 18) was obtained to collect tissue for research in all autopsy cases and in all surgical cases collected since 2010, with explicit consent for use for next generation sequencing since 2016. For surgical cases prior to 2010 or where the consent did not explicitly state the tissue would be used for next generation sequencing, for deceased patients, waiver of consent to use the tissue for this purpose was granted by the Hospital for Sick Children Research Ethics Board.<br>After obtaining consent, tumour and normal brain samples were taken at surgery or postmortem from pediatric patients diagnosed with high-grade glioma presenting to The Hospital for Sick Children and immediately snap frozen in liquid nitrogen then stored at -80 C. We analysed all available samples with material of sufficient quality/quantity, so there were no/minimal self-selection biases.
Ethics oversight	The Hospital for Sick Children Research Ethics Board (#1000055059)

Note that full information on the approval of the study protocol must also be provided in the manuscript.
